# Maize *ZmbZIP33* Is Involved in Drought Resistance and Recovery Ability Through an Abscisic Acid-Dependent Signaling Pathway

**DOI:** 10.3389/fpls.2021.629903

**Published:** 2021-04-01

**Authors:** Liru Cao, Xiaomin Lu, Guorui Wang, Qianjin Zhang, Xin Zhang, Zaifeng Fan, Yanyong Cao, Li Wei, Tongchao Wang, Zhenhua Wang

**Affiliations:** ^1^Grain Crops Research Institute, Henan Academy of Agricultural Sciences, Zhengzhou, China; ^2^National Key Laboratory of Wheat and Maize Crop Science, College of Agronomy, Henan Agricultural University, Zhengzhou, China; ^3^State Kay Laboratory of Agro-biotechnology and Key Laboratory of Pest Monitoring and Green Management-MOA, China Agricultural University, Beijing, China

**Keywords:** maize, ABA pathways, drought resistance, recovery ability, transcriptome

## Abstract

Analyzing the transcriptome of maize leaves under drought stress and rewatering conditions revealed that transcription factors were involved in this process, among which *ZmbZIP33* of the ABSCISIC ACID-INSENSITIVE 5-like protein 5 family was induced to significantly up-regulated. The functional mechanism of *ZmbZIP33* in Abscisic acd (ABA) signaling pathway and its response to drought stress and rewatering has not been studied yet. The present study found that ZmbZIP33 contains a DNA-binding and dimerization domain, has transcriptional activation activity, and is highly homologous to SbABI1,SitbZIP68 and OsABA1. The expression of *ZmbZIP33* is strongly up-regulated by drought, high salt, high temperature, and ABA treatments. Overexpression of *ZmbZIP33* remarkably increased chlorophyll content and root length after drought stress and rewatering, and, moreover, cause an accumulation of ABA content, thereby improving drought resistance and recovery ability in *Arabidopsis*. However, silencing the expression of *ZmbZIP33* (BMV-ZmbZIP33) remarkably decreased chlorophyll content, ABA content, superoxide dismutase and peroxidase activities, and increased stomatal opening and water loss rate compared with BMV (control). It showed that silencing *ZmbZIP33* lead to reduced drought resistance and recovery ability of maize. ABA sensitivity analysis found that 0.5 and 1 μmol/L treatments severely inhibited the root development of overexpression *ZmbZIP33* transgenic *Arabidopsis*. However, the root growth of BMV was greatly inhibited for 1 and 5μmol/L ABA treatments, but not for BMV-ZmbZIP33. Subcellular localization, yeast two-hybrid and BIFC further confirmed that the core components of ABA signaling pathways ZmPYL10 and ZmPP2C7 interacted in nucleus, ZmPP2C7 and ZmSRK2E as well as ZmSRK2E and ZmbZIP33 interacted in the plasma membrane. We also found that expression levels of *ZmPYL10* and *ZmSRK2E* in the BMV-ZmbZIP33 mutant were lower than those of BMV, while *ZmPP2C7* was the opposite under drought stress and rewatering. However, expression of *ZmPYL10* and *ZmSRK2E* in normal maize leaves were significantly up-regulated by 3–4 folds after drought and ABA treatments for 24 h, while *ZmPP2C7* was down-regulated. The *NCED* and *ZEP* encoding key enzymes in ABA biosynthesis are up-regulated in overexpression *ZmbZIP33* transgenic line under drought stress and rewatering conditions, but down-regulated in BMV-ZmbZIP33 mutants. Together, these findings demonstrate that *ZmbZIP33* played roles in ABA biosynthesis and regulation of drought response and rewatering in *Arabidopsis* and maize thought an ABA-dependent signaling pathway.

## Introduction

Droughts have a severe and negative impact on maize yield. Coupled with the shortage of fresh water resources in China, the effects of drought, e.g., loss of maize yields, lowered quality of agricultural products, degradation of agricultural resources, and damage to the environment, are more severe than of other natural disasters (Keshan Peng and Hu, 2002). In order to alleviate the effects of insufficient water resources and drought on maize yields, the discovery and innovation of drought-resistant and water-saving genetic resources is the key to the development of water-saving agriculture. Genetic recombination through genetic engineering has been used to create new maize varieties with improved water-saving abilities, drought tolerance, and higher yields. In recent years, researchers have used high-throughput sequencing technology and genetic modification methods to explore molecular resources for drought resistance and water saving. These have been used to generate high-quality drought-resistant germplasm resources, and moreover, drought-resistance and water-saving genes have been used to improve the water saving abilities of plants (Zhang, 2004). Fernandes et al. used the transcriptome platform to sequence the maize B73 inbred line under drought stress and found that 40 were down-regulated genes and 17 were up-regulated genes in response to drought stress (Burgess, 2012). The results of *Arabidopsis* transcriptome sequencing combined with cell morphology analysis revealed the regulatory mechanism of abscisic acid (ABA) in Arabidopsis guard cells under drought stress (Wang et al., 2011).

The plant hormone ABA is a key regulator of responses to various stresses such as drought, high temperature, salinity, transpiration rate, and other water-saving and drought-resistant mechanisms (Claeys, 2013). Stress avoidance and stress tolerance are distinct mechanisms employed by plants to cope with low-water conditions, both consist of ABA-dependent and ABA-independent mechanisms (Claeys, 2013). Numerous studies have extensively showed that plants accumulate ABA under drought conditions, thereby controlling stomatal closure and saving water (Murata et al., 2015). In the ABA-dependent pathway, osmotic receptors on plant cell membranes directly trigger a second signaling system that transmits information without being mediated by ABA. The dehydration responsive element (DRE)-binding protein (DREB) genes contain a 9-bp forward repeat (TACCGACAT). The core sequence of CCGAC plays a key role in the co-induced pathways of drought and low temperature stress response (Liu et al., 1998). Genes in the ABA-dependent signaling pathway are primarily regulated by transcription factors (TFs) and cis-acting element ABRE ABA-responsive elements. ABA binds with receptors of the PYR/PYLs family. Alternatively, PYR/PYL receptors are bound by protein phosphatase 2C (PP2C) forming a PYR/PYL-PP2C complex (Ma et al., 2009; Park et al., 2009; Santiago et al., 2009). Protein phosphatase 2C binds with sucrose-nonfermenting kinase1-related protein kinases, SnRK2.6/SnRK2E/SnRK2D, and SnRK2.3/SnRK2I, to negatively regulate ABA-mediated responses (Mehrotra, 2014). SnRK positively regulates the activation of ABSCISIC ACID-INSENSITIVE 5-like protein 5 in *Triticum aestivum* (Johnson, 2002), and binds to cis-acting elements to induce downstream gene expression. The bZIP subfamily is functionally related to ABA pathway (Mittal et al., 2014). The bZIP dimer specifically binds to the promoter and up-regulates the genes encoding proline dehydrogenase under hypotonic stress. (Schutze et al., 2008). BZIP genes of *AtbZIP36, AtbZIP37*, and *AtbZIP38* are up-regulated in response to drought, salt stress, and following by ABA treatment in *Arabidopsis* (Uno et al., 2000). Hossain et al. found that bZIP genes of *OsbZIP12* and *OsbZIP46* are also strongly up-regulated by the stresses mentioned above, as well as by phytohormone treatment (Hossain et al., 2010). In soybean, the overexpression of *GmbZIP1*, a member of the AREB family, that is involved in stomatal closure, leads to improved abiotic stress tolerance (Gao et al., 2011).

Previous studies have shown that ZmbZIP33 (ABSCISIC ACID-INSENSITIVE 5-like protein 5,GenBank accession number: ACF82064.1) may be one of the key genes in response to drought stress (Cao et al., 2019),and that its *Arabidopsis* homologous gene, *At3g19290*,responds to drought and salt stress through an ABA-dependent signaling pathway(Uno et al., 2000). To date, the stress resistance and regulatory mechanism of *ZmbZIP33* in maize have not reported. Therefore, this study analyzed the expression pattern of *ZmbZIP33* in response to a variety of abiotic stresses and ABA induction, and further verified its drought resistance and recovery ability by overexpression and virus-induced gene silencing (VIGS) transgenic technology. Further, its mechanism of its action was explored using yeast two-hybrid system and bimolecular fluorescence complementation technology. The results from our study provide excellent molecular resources for the genetic improvement of drought tolerance in maize, and further establish a theoretical foundation for drought-resistant germplasms, which has an important practical significance for improving the water saving strategy of maize and ensuring the safety of maize production in the future.

## Materials and Methods

### Plant Growth Conditions and Drought Treatment

“Yu882” (*Zea mays L*., inbred line) was germinated for 5 days, and the seedlings were transferred to half-strength modified Hoagland's nutrient solution with growth conditions at 28°C in a 14/10-h photoperiod. Three-leaf-stage maize were transferred into nutrient solution containing 20% polyethylene glycol 6000 (PEG). Leaves of PEG stress and non-stress were harvested at 60 and 96 h and after 3 days recovery (denoted as T60, T96, TR3d and CK60, CK 96, CK R3d, respectively). The samples at these time points were subjected to RNA sequencing (RNA-Seq). The second experiment, we transferred seedings of three-leaf stage to nutrient solution containing 20% PEG _6000_, NaCl (200 mmol/L), ABA (5 μmol/L) and 37°C conditions. The leaves of maize were collected at 0, 4, 12, 24, 36 h and stored at −80°C. Each treatment had three biological replicates.

The plants of maize inbred line Va35 were grown in an artificial climate incubator with 20°C/18°C (photoperiod: 16 h light/8 h dark). Tobacco was grown in an artificial climate chamber at 24°C/22°C (photoperiod: 14 h light /10 h dark).

### Transcriptome Sequencing Using Illumina HiSeq™ 2500 Platform

Total RNA was extracted using TRIzol reagent (Invitrogen, Carlsbad, CA, USA). A Qubit Fluorometer (Invitrogen) was used to measure RNA concentration, and an Agilent 2100 Bioanalyzer (Agilent, Palo Alto, California, USA) was used to verify RNA quality. The minimum RNA integration value was 8. After total RNA is extracted using TRIzol, RNA molecules with a size of 18–30 nt can be enriched by polyacrylamide gel electrophoresis (PAGE).

Use the Illumina HiSeq™2500 platform to reverse transcription of the enriched mRNA, amplify and sequence by polymerase chain reaction. High-quality clean reads are obtained by removing those containing adaptors and those containing more than 10% of unknown nucleotides (N) and more than 50% of low-quality (Q value ≤ 20) bases. Lastly, ribosome RNA (rRNA) mapped reads were removed through mapping reads to the rRNA database using the short reads alignment tool Bowtie2. The remaining reads are used for transcriptome assembly, and the raw data is compared with the maize inbred line B73V3 database and gene annotation. Use Blast2GO to analyze the pathway of these genes.

To identify differentially expressed genes (DEGs) samples or groups, the R package (http://www.r-project.org/) was used. We identified genes with |log2 (fold change)| ≥ 2 and a false discovery rate (FDR) <0.05 in a comparison as significant differentially expressed genes. Use R language heat map software for differential gene expression enrichment. Calculate the correlation coefficient between genes based on the expression of genes. We imported a correlation coefficient greater than 0.75 into Cytoscape software 3.71 version [ion3.7.1 (https://cytoscape.org)] with to make gene co-expression network to screen key node genes.

### Isolation of RNA and Quantitative Real-Time PCR Analysis

Total RNA was extracted using Total RNA Extraction Reagent (YEASEN, Shanghai, China). Hifair® II 1st Strand cDNA Synthesis SuperMix (YEASEN, Shanghai, China) was applied to synthesize first-strand cDNA. The quantitative real-time PCR (qRT-PCR) was carried out using hieff® qPCR SYBR® Green Master Mix (YEASEN, Shanghai, China). According to the corresponding sequences, we designed gene-specific primers using Vector NTI11.5, as shown in Supplementary Table 2. Each gene was analyzed with three technical replicates. The relative expression level (2^−ΔΔCt0h^) in the control plants was normalized to 1.

### Transactivation Activity in Yeast

The CDS of *ZmbZIP33* was amplified by PCR using a pair of gene-specific primers (Supplementary Table 2), and then ligated to pGBKT7 vector digested by EcoRI/BamHI. PGBKT7-ZmbZIP33 construct, empty pGBKT7 vector (negative control) and pGBKT7-53 plus pGADT7-T (positive control) were transformed into yeast strain AH109 by lithium acetate-mediated method, respectively. The transformed yeast cells were examined on SD/-Trp and SD/-Trp/-His/-Ade/X-a-Gal medium at 30°C for 5 days.

### Determination of Subcellular Localization

The complete open reading frames (ORF) of Z*mbZIP33, ZmSRK2E* and *ZmPP2C7* were PCR-amplified, and then constructed into pMDC83-GFP vector. The primers are shown in Supplementary Table 2. The constructed ZmbZIP33-GFP, ZmSRK2E-GFP, ZmPP2C7-GFP, and pMDC83-GFP were transferred into Nicotiana benthamiana leaves by Agrobacterium infection. Two days later, the fluorescence of tobacco leaves was observed under a confocal microscope of Zeiss LSM700 (Zeiss, Jena, Germany).

### Yeast Two-Hybrid (Y2H) Assays

The ORF of *ZmbZIP33* and *ZmPP2C7, ZmSRK2E* and *ZmPYL10* genes were cloned into vectors pGBKT7 and pGADT7, respectively. Subsequently, the pGBKT7-ZmPP2C7+pGADT7-ZmPYL10,PGBKT7-ZmPP2C7+pGADT7-ZmSRK2E, pGBKT7-ZmbZIP33+pGADT7-ZmSRK2E and pGADT7-T+ pGBKT7-Lam (negative control), pGADT7-T+pGBKT7-53 (positive control) were introduced into the yeast strain using the high-efficiency lithium acetate transformation procedure (Xiao et al., 2011). The primers are shown in Supplementary Table 2.

### Bimolecular Fluorescence Complementation (BIFC)

The ORF of *ZmbZIP33* and *ZmPP2C7, ZmSRK2E* and *ZmPYL10* genes were cloned into vectors of pSAT4-nEYFP and pSAT4-cEYFP, respectively. The pSAT4-nEYFP-ZmPP2C7 +pSAT4-cEYFP-ZmPYL10,pSAT4-nEYFP-ZmPP2C7+pSAT4-cEYFP-ZmSRK2E, pSAT4-nEYFP-ZmbZIP33+pSAT4-cEYFP-ZmSRK2E, pSAT4-cEYFP-ZmPYL10+nEYFP, pSAT4-nEYFP-ZmPP2C7+cEYFP,pSAT4-cEYFP-ZmSRK2E+nEYFP and pSAT4-nEYFP-ZmbZIP33+cEYFP were then introduced into Nicotiana benthamiana leaves by Agrobacterium infection, respectively. Two days later, SP5 Meta confocal laser microscope (Leica, Germany) was used to observe the fluorescence signal, and yellow fluorescence signal represents an interaction between the two proteins. The primers are shown in Supplementary Table 2.

### *Arabidopsis* and Maize Transformed Plants and Drought Resistance and Resilience Analysis

The *ZmbZIP33* was cloned into pFGC5941 vector (with bar label) using specific primers (Supplementary Table 2), and then introduced into *Arabidopsis* by agrobacterium. Three independent homozygous lines, L1, L2, and L3 were selected. Transgenic *Arabidopsis* line with overexpression of *ZmbZIP33* and Wild type (WT,Col) seedlings grown on 1/2 MS culture medium (MS) for 1 week. Then transferred to MS medium with 150 and 250 mM of mannitol. One week later, the root length of transgenic lines and Col (WT) *Arabidopsis* was observed. Simultaneously, transgenic lines and Col seedlings were transplanted into the soil with portions of sand, compost, and perlite (3: 3: 1, respectively) for 20 days in a climate chamber at 22°C with 60% of relative humidity in a 16-h/8-h day/night cycle. Drought stress was applied to transgenic lines and Col plants for 4 and 6 days, then watered for 5 days.

The specific fragments of 180 bp in the ORF region of *ZmbZIP33* were selected for designing primers (Supplementary Table 2). Virus-induced gene silencing (VIGS) was used to silence ZmbZIP33.BMV-GFP is the vector for transiently silencing gene expression. The *ZmbZIP33* gene was isolated from Va35 maize and loaded into the BMV-GFP vector to obtain the ZmbZIP33-BMV fusion. Then, BMV-ZmbZIP33 and empty BMV-GFP were introduced into Agrobacterium tumefactions strain GV3101. The Agrobacterium liquid of BMV-ZmbZIP33 and empty BMV-GFP were mechanically inoculated onto the leaves of A. tumefaciens-infiltrated N. benthamiana plants at 24 h post-infiltration, as described previously(Chen et al., 2017). Three days later, tobacco leaves were taken for virus extraction according to a previous method (Chen et al., 2017). The extracted BMV-ZmbZIP33 and BMV-GFP tobacco virus were infected into a second leaf of Va35 maize,and cultured in a 20°C light incubator for 6 days with a light-transmitting plastic cover. The plastic cover was subsequently removed and placed in a 22°C light incubator. BMV-GFP serves as a wild-type control, hereinafter referred to as BMV. The expressions of *ZmbZIP33* were detected 7 days after virus infection on *ZmbZIP33* mutant (BMV-ZmbZIP33) and BMV (Supplementary Figure 4B), and BMV-ZmbZIP33 and BMV plants were irrigated with 40% of PEG for 0, 4, and 6 days, respectively, and then watered for 4 days.

The activities of superoxide dismutase (SOD) and peroxidase (POD) were measured according to Azevedo et al. (1998). Chlorophyll content was measured according to Lakra et al. (2015). ABA content was measured using an enzyme-linked immunosorbent assay (ELISA) under drought stress and rewatering, as previously described (Yang et al., 2001). Leaves at three-leaf stage of BMV-ZmbZIP33 and BMV were detached, and the water loss rates of each lines were calculated within 5 h.

### ABA Sensitivity of *Arabidopsis* and Maize Transformed Plants

Three independent homozygous lines, L1, L2, and L3 were selected. Transgenic lines and WT (Col) seedlings were grown on MS medium with 0, 0.5 and 1 μmol/L of ABA. Ten days later, the sensitivities of transgenic lines and Col to ABA were observed.

BMV-ZmbZIP33 and BMV seedings were germinated for 5 days, and the seedlings were transferred to half-strength modified Hoagland's nutrient solution. Three-leaf-stage maize were transferred to nutrient solution containing 0, 1, and 5 μmol/L ABA. Five days later, the sensitivities of BMV-ZmbZIP33 and BMV to ABA were observed.

### Scanning Electron Microscopy (SEM) Images of Stomata

BMV-ZmbZIP33 and BMV plants were irrigated with 40% of PEG for 0,4 and 6 days, respectively, and then watered for 4 days. The leaves stomata image of BMV-ZmbZIP33 and BMV were obtained by scanning electron microscopy (XL-30ESEM, PHILIPS, Netherland).

## Results

### Phenotype Changes of Maize Under Drought Stress and Rewatering in Maize

When using 20% Polyethylene glycol 6000 (PEG) to simulate drought stress, it was found that compared with CK (control), the maize leaves and particularly the tips began to wilt after 60 h. After 96 h, the leaves were severely and the leaf tips had begun to die. After 3 days of rewatering, the leaves gradually expanded, however, the growth was worse than that of CK (Supplementary Figure 1), indicating that the maize had a partial compensation effect after rewatering. According to the phenotypic results, we selected 60 h, 96 h, and 3 days leaves for high-throughput sequencing (RNA-Seq). The sequencing data has been published in NCBI, and the accession number is PRJNA477643 (https://www.ncbi.nlm.nih.gov/sra/?term=SRA729707).

### Study of Transcription Factors in Maize Transcriptome Sequencing Under Drought Stress and Rewatering

**Figure 1:**
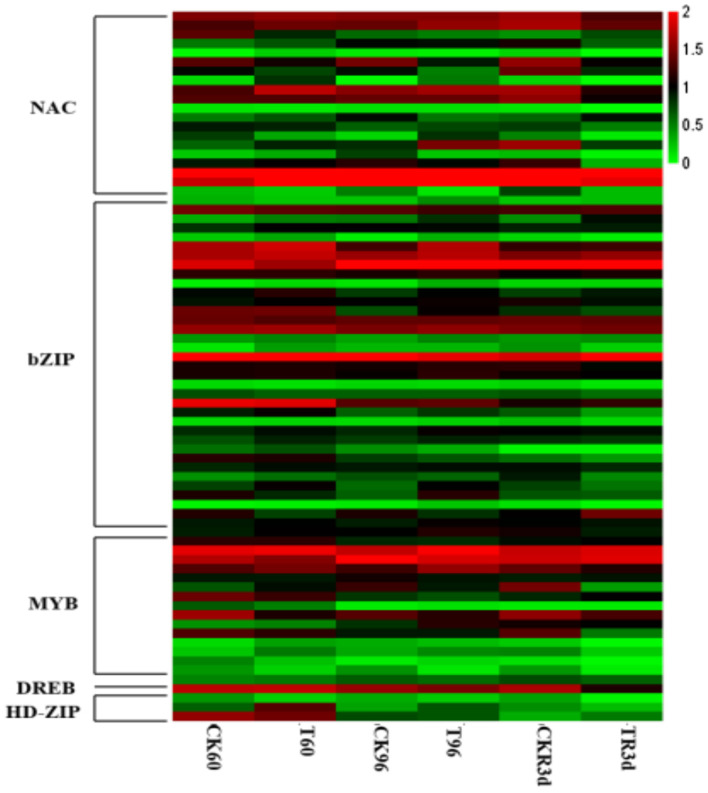
The differential expression transcription factors in maize transcriptome sequencing under drought stress and rewatering.

Functional annotation was performed on the DEGs obtained by RNA-Seq, and MYB/MYC, bZIP, NAC, HD-ZIP and DREB families transcription factors (TFs) were enriched (Supplementary Figure 3). All TFs except *ZmbZIP35, ZmMYB1, ZmMYB2, ZmMYB11, ZmNAC6, ZmNAC7, ZmNAC12, ZmNAC16*, and *ZmNAC20* increased within 96 h of drought stress, however their expression levels decreased after 3 days of rewatering compared with CK (Figure 1, Supplementary Table 1).

### The Gene Co-expression Network Analysis of TFs

**Figure 2:**
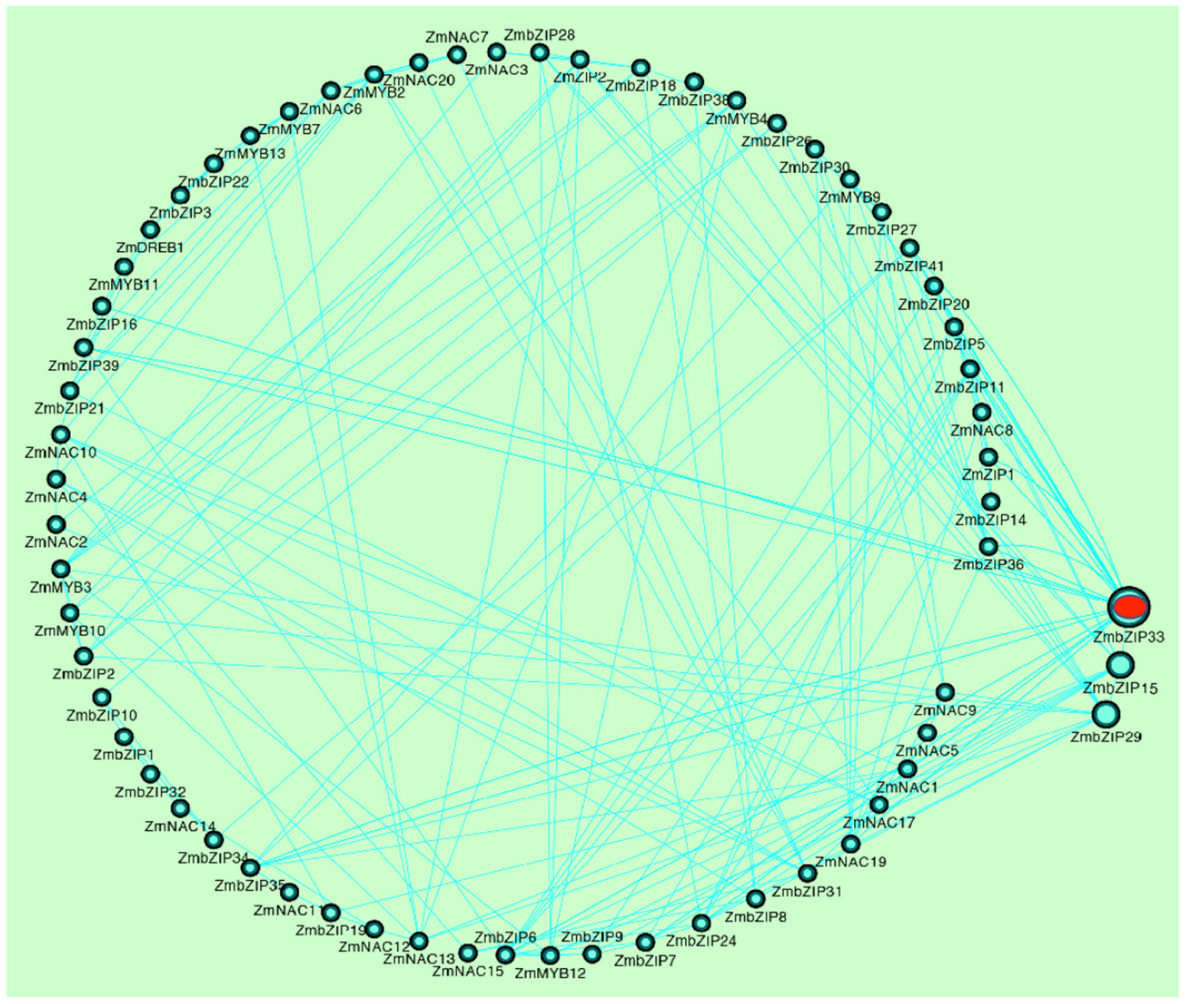
The gene co-expression network of TFs. The gene co-expression network between 77 TFs was constructed by Cytoscape software (correlation coefficient is greater than 0.75). The straight line represents a correlation between genes. The circle represents the TFs, and the larger the circle, the more connected to other TFs.

By analyzing the correlation coefficients between the expression levels of these TFs, a co-expression network was constructed. The core genes that respond to drought stress and rewatering were further screened by the co-expression network. As shown in fig.2, *ZmbZIP33, ZmbZIP15*, and *ZmbZIP19* had a higher correlation with more TFs, indicating that these three genes are at the core of the co-expression network. We speculate that these three genes, especially *ZmbZIP33*, play an important role in response to drought stress and rewatering. The expression of *ZmbZIP33* was significantly up-regulated at 60 and 96 h under drought stress, and down-regulated after rewatering. It is worthwhile to further explore the drought tolerance and mechanism of *ZmbZIP33*.

### The Expression of *ZmbZIP33* Under Abiotic Stresses and ABA Treatment

**Figure 3:**
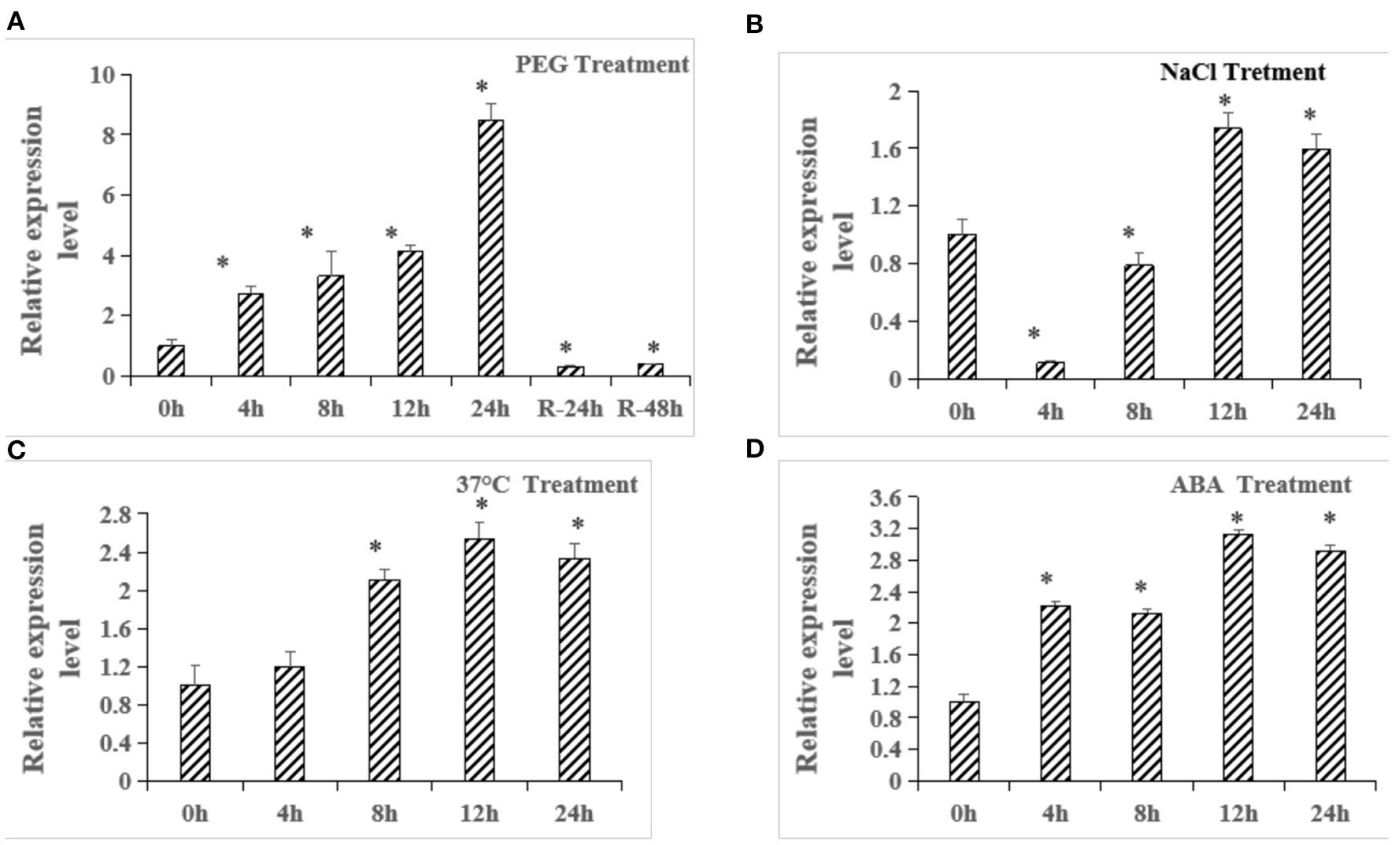
Quantitative RT-PCR analysis of *ZmbZIP33* in maize leaves under abiotic stresses and ABA treatment. **(A)** Relative expression of *ZmbZIP33* gene under 20% PEG _6000_ treatment at 0, 4, 8, 12, 24 h and rewatering at 24 h (R-24 h) and 48 h (R-48 h). **(B–D)** Relative expression of *ZmbZIP33* gene under NaCl (200 mmol/L), 37°C and ABA (5 μmol/L) treatment at 0, 4, 8, 12, and 24 h. Mean values and standard errors (bar) were shown from three independent experiments. Independent *t*-tests for equality of means demonstrated that there was significant difference between CK and treatment (*P value < 0.05). The followings are the same.

**Figure 4:**
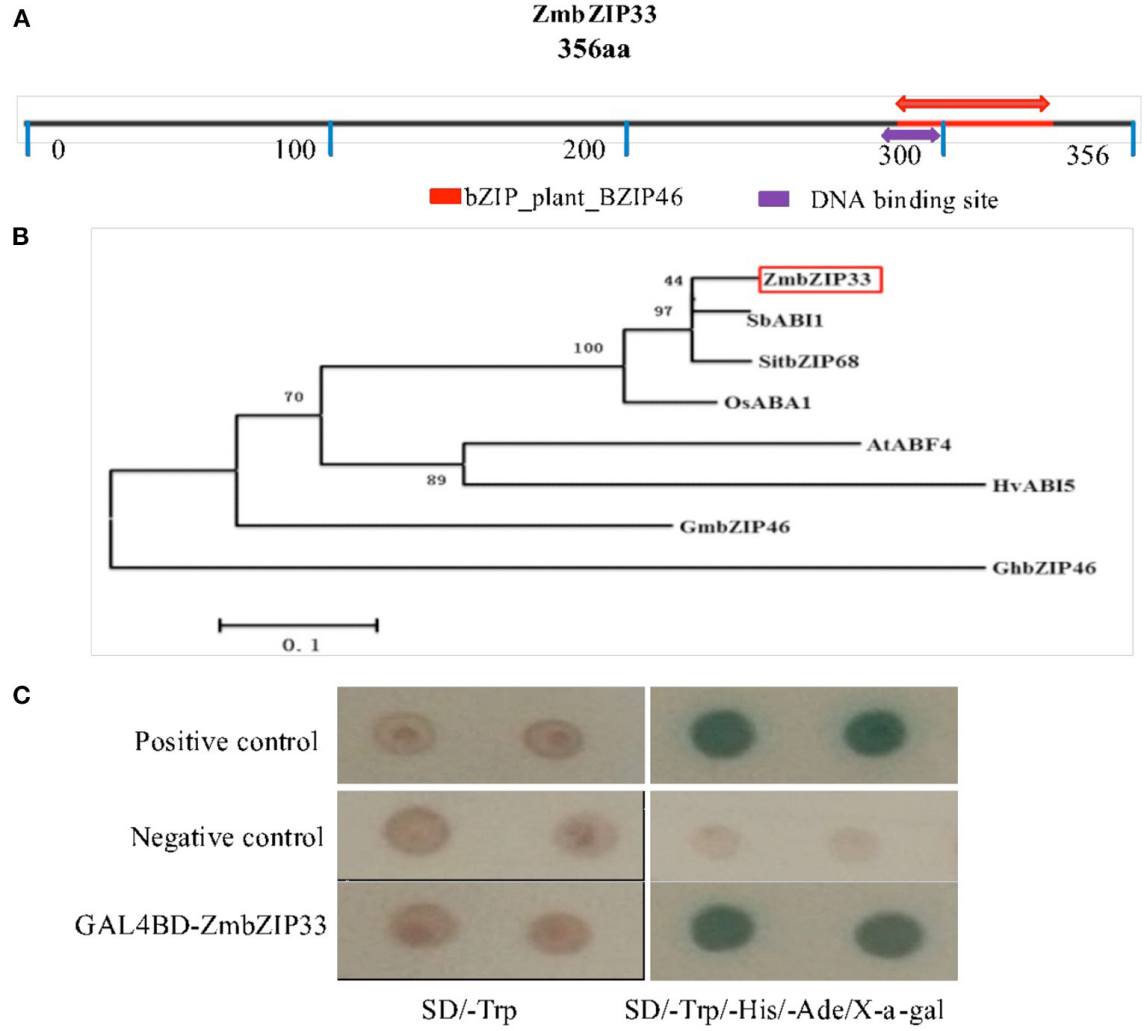
Characterization of *ZmbZIP33* gene. **(A)** The length of amino acid encoded by *ZmbZIP33* gene and its domain. **(B)** Relationships between ZmbZIP33 and its homologs. Phylogenetic tree was generated using MEGA6 version **(C)** The construct pGBKT7-*ZmbZIP33* was transformed into yeast strain AH109 and checked on SD/Trp and SD/Trp-/His-/Ade-/X-α-gal plates. pGBKT7-53 and pGADT7-T were used as a positive control, and pGBKT7 vector was used as a negative control.

Based on the results in Figures 1, 2, we speculate that *ZmbZIP33* is a key gene in response to drought stress and rewatering. Using qRT-PCR to further analyze the expression patterns of *ZmbZIP33* in response to drought and other abiotic stresses and ABA treatment. As shown in **Figure 4**, expressions of *ZmbZIP33* in leaves were significantly up-regulated by PEG, NaCl, 37°C and ABA. *ZmbZIP33* gene is strongly induced by PEG and reached a maximum 24 h, following this, the expression level dropped sharply 24 h after rewatering (Figure 3A). By contrast, *ZmbZIP33* transcripts declined to their highest at 12 h following NaCl treatment (Figure 3B). Furthermore, it was found that *ZmbZIP33* mRNA accumulates at 4 h and reached its highest level at 12 h following 37°C and ABA treatments (Figures 3C,D).

### Characterization of *ZmbZIP33* Gene

The CDS of *ZmbZIP33* (GenBank accession number: ACF82064.1) was isolated using reverse-transcription PCR (RT-PCR) from the cDNA library of the “Yu 882” maize inbred line. The nucleotide sequence of *ZmbZIP33* contains a 1,071 bp open reading frame that encodes a protein of 356 amino acids including a bZIP_plant_BZIP46 domain (Figure 4A), which is a DNA-binding and dimerization domain. *ZmbZIP33* encodes ABSCISIC ACID-INSENSITIVE 5-like protein 5. This family protein is mainly involved in ABA-activated signaling pathway and positive regulation of transcription. A protein phylogenetic analysis showed that ZmbZIP33 is closely related to SbABI1, SitbZIP68 and OsABA1 (Figure 4B). Studies have shown that SbABI1 and OsABA1 proteins respond to abiotic stress by participating in the ABA pathway (Kagaya et al., 2002; Kobayashi et al., 2005). The present study have confirmed that *ZmbZIP33* actively responded to exogenous ABA treatment. Therefore, *ZmbZIP33* gene may also have similar functions to homologous genes, responding to drought stress and relying on ABA pathways.

First, we verified whether *ZmbZIP33* has transcriptional activity. The open-reading frame of *ZmbZIP33* was fused with the GAL4 DNA-binding domain of the pGBKT7 vector. The pGBKT7-ZmbZIP33 construct was transformed into yeast strain AH109. As shown in Figure 4C, the yeast cells harboring negative control grew on the SD/-Trp medium only and did not exhibit α-galactosidase activity. In contrast, the transformants containing pGBKT7-ZmbZIP33 and the positive control grew well on the SD/-Trp medium and SD/-Trp/-His/-Ade/X-α-Gal medium. These results suggested that *ZmbZIP33* had transcriptional activity in yeast.

### Overexpression of *ZmbZIP33* in Transgenic *Arabidopsis* Enhanced Drought Resistance and Recovery Ability

The coding sequence of *ZmbZIP33* was fused with the CaMV 35S promoter and T3 generation transgenic lines were obtained after the resulting construct was transfected into *Arabidopsis*. The expression level of *ZmbZIP33* in overexpression lines were significantly higher than that of Col (WT) (Supplementary Figure 4A). The segregation of empty vector and *bZIP33* transgenes and test goodness of fit for Medellian patterns were also investigated (Supplementary Table 3). The results showed that three transgenic *Arabidopsis* lines of *ZmbZIP33* (L1–L3) were successfully selected.

**Figure 5:**
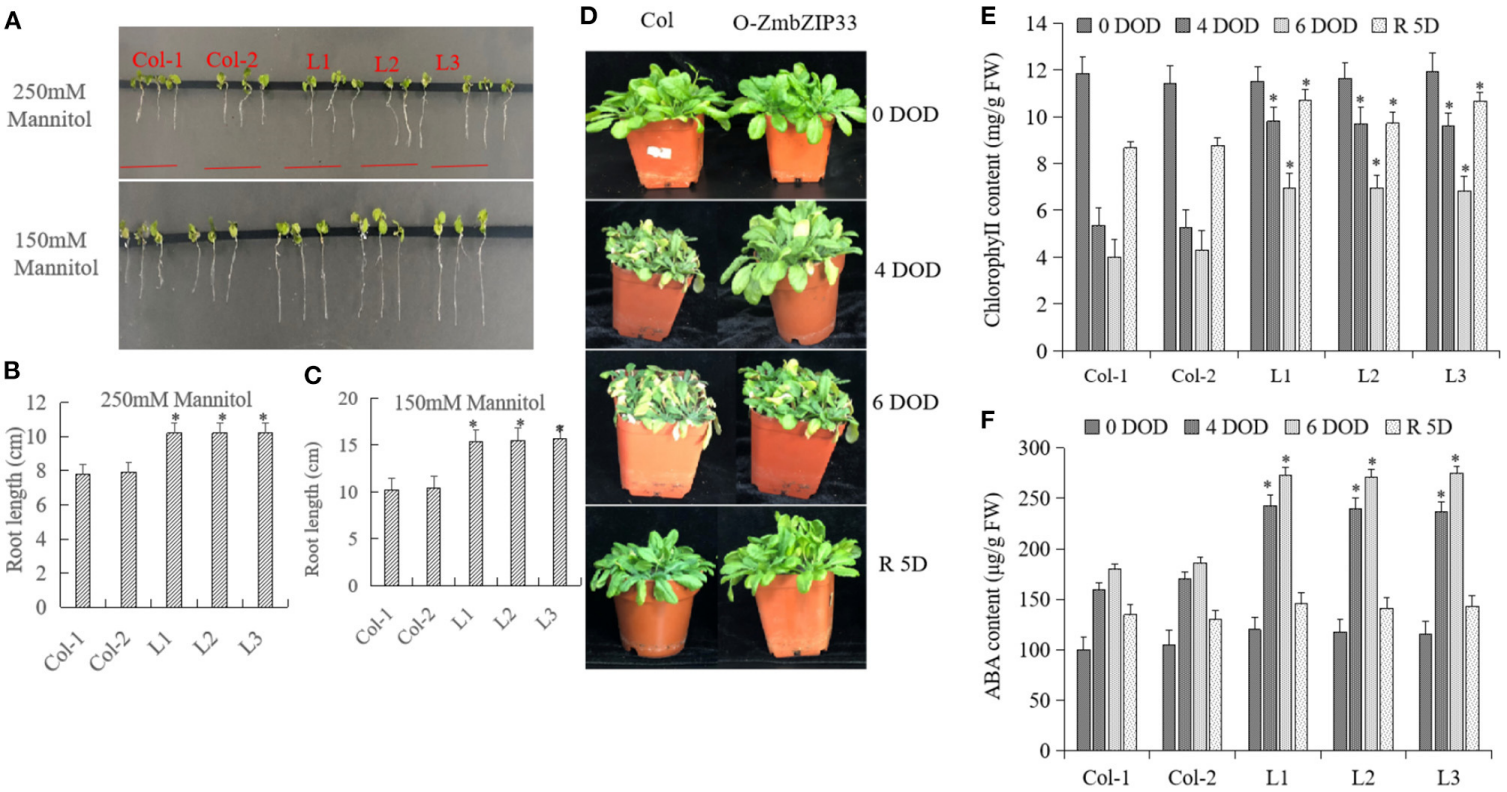
The root length and the contents of chlorophyll and ABA in overexpression *ZmbZIP33* transgenic *Arabidopsis*. **(A)** Col is the wild type (WT), L1–L3 are *ZmbZIP33* transgenic lines. Five-days old seedlings were transferred and grown for 3 days on MS medium supplemented with 250 and 150 mM of Mannitol. **(B/C)** The root lengths of *ZmbZIP33* transgenic *Arabidopsis* and Col-0 in 250 and 150 mM of Mannitol. **(D)** Col and O-ZmbZIP33 plants were exposed for 4 and 6 days of drought (DOD). The plants were then rewatered for 5 days (R 5D) and photographed. **(E/F)** The contents of chlorophyll and ABA of Col and L1–L3 leaves were measured under drought stress and rewatering. Mean values and standard errors (bar) were shown from five independent experiments. Independent *t*-tests for equality of means demonstrated that there was significant difference between control and treatment (*P value < 0.05).

Col and transgenic *Arabidopsis* lines were treated with 150 and 250 mM of mannitol to simulate drought stress (Figure 5A). There was no difference between the phenotype of Col and transgenic *Arabidopsis* before drought treatment. Following 1 week of treatment, it was found that the roots of the transgenic lines were longer than those of the Col at both mannitol concentrations (Figures 5B,C), indicating that *ZmbZIP33* overexpression increased drought resistance of *Arabidopsis*. After 4 and 6 days of drought exposure, the transgenic *Arabidopsis* lines grew better than Col. After rewatering for 5 days, both Col and transgenic *Arabidopsis* lines recovered, and the transgenic *Arabidopsis* lines recovered better than the Col (Figure 5D). The leaves chlorophyll contents of the leaves of L1–L3 transgenic lines were significantly higher than that of Col after drought stress and rewatering (Figure 5E). This indicated that the overexpression of *ZmbZIP33* improved drought resistance and recovery ability of *Arabidopsis*. In addition, we found that the difference in ABA content between Col and L1–L3 transgenic lines was small before stress (0 DOD), however after 4 and 6 days of drought stress, the ABA contents in transgenic lines were significantly higher than Col. Interestingly, after 5 days of rewatering, the ABA contents in L1–L3 transgenic lines dropped rapidly and approached the level found in the Col (Figure 5F).

### Transiently Silencing of *ZmbZIP33* Inhibits Stomatal Closure and Increases Water Loss Under Drought Stress and Rewatering

**Figure 6:**
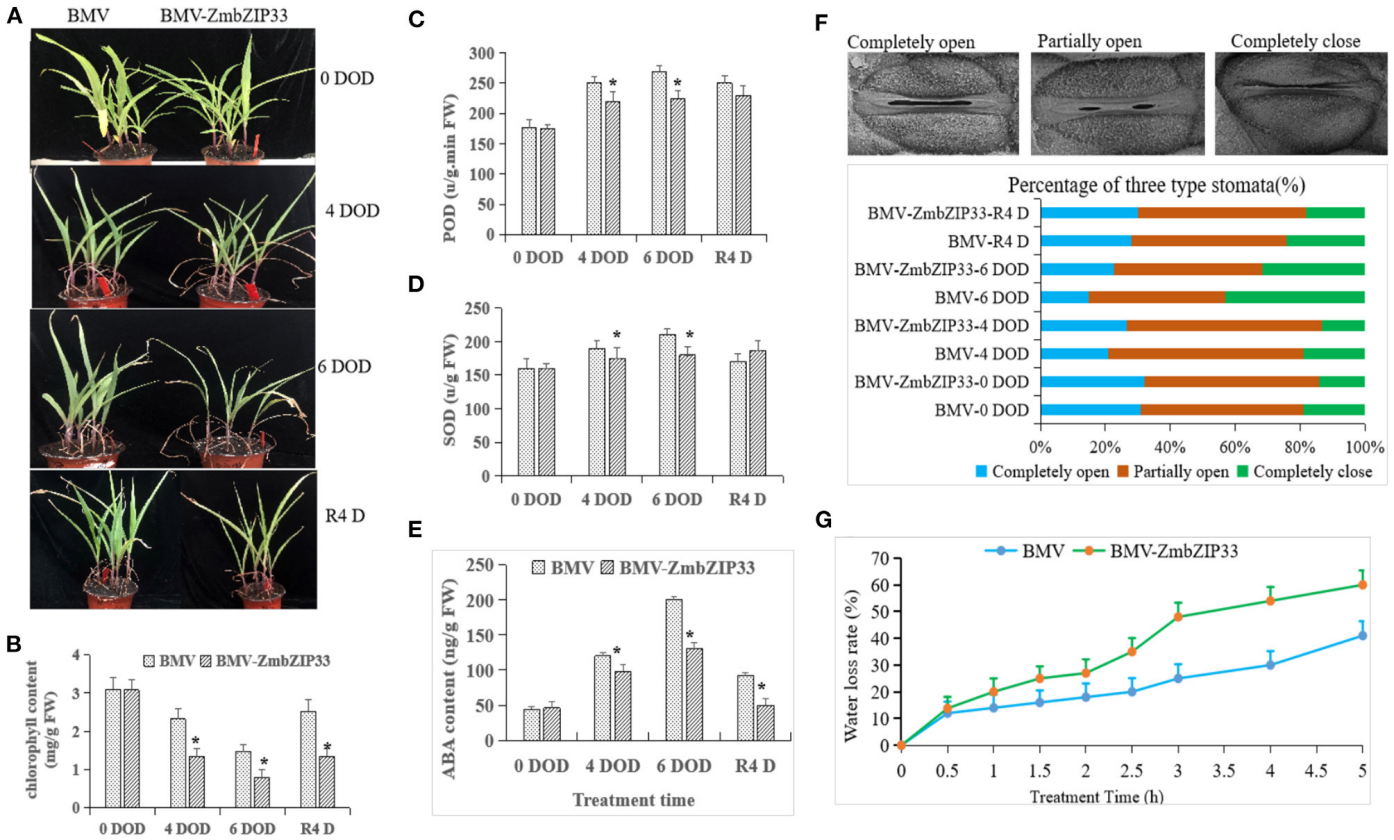
Analysis of ABA sensitivity and drought resistance of *ZmbZIP33* under drought stress. **(A)** Forty percent of PEG stress on BMV and BVM-ZmbZIP33 maize seedlings for 4 and 6 days (DOD). The plants were then rewatered for 4 days (R4 D) and photographed. **(B–E)** Chlorophyll content, SOD activity, POD activity, and ABA content of BMV and BVM-ZmbZIP33 leaves were measured under drought stress and rewatering. **(F)** Scanning electron microscopy images of three levels of stomatal opening in BMV and BMV-ZmbZIP33. Bar = 20 μm. **(G)** Water loss assays for the leaves of BMV and BMV-ZmbZIP33 transgenic line were performed within 5 h. Mean values and standard errors (bar) were shown from five independent experiments. Independent *t*-tests for equality of means demonstrated that there was significant difference between control and treatment (*P value < 0.05).

Virus-induced gene silencing (VIGS) was used to silence *ZmbZIP33* in order to study the drought resistance and recovery ability of maize (Figure 4B). Next, BMV and BMV-ZmbZIP33 mutant were subjected to drought stress for 4 and 6 days, respectively. It was found that BMV and BMV-ZmbZIP33 leaves became curled up, wilted and lost their green color, and gradually the leaf tips withered. Additionally, the BMV seedlings grew better than the BMV-ZmbZIP33 seedlings. Following 4 days of rewatering, BMV resumed growth, but BMV-ZmbZIP33 growth did not return to normal level (Figure 6A). The chlorophyll content of BMV leaves was significantly higher than that of BMV-ZmbZIP33 leaves after drought stress and rewatering (Figure 6B). After drought stress, the activity of POD and SOD in BMV and BMV-ZmbZIP33 leaves increased and these effects were more pronounced for BMV than for BMV-ZmbZIP33 (Figures 6C,D), resulting in a decrease in drought resistance and recovery ability of maize.

The accumulation of ABA content promotes stomatal closure and reduces water evaporation. To further study the role of *ZmbZIP33* in regulating water saving, we tested ABA content, stomatal closure and water loss tests under drought stress and rewatering in BMV and BMV-ZmbZIP33.ABA concentration was below detection level before drought in BMV and BMV-ZmbZIP33,whereas, in response to drought, ABA content significantly increased in both genotypes and decreased after rewatering. Interestingly, ABA concentration in BMV was significantly higher than that in BMV-ZmbZIP33 under drought stress (Figure 6E). Measurements of stomatal movements in the leaves showed that completely closed stomata number were less and completely opened stomata number were more in BMV-ZmbZIP33 compared with BMV plants (Figure 6F). Measurements of the water loss rate of leave showed that BMV-ZmbZIP33 plants lost more water than BMV plants (Figure 6G). These results suggested that silencing the expression of *ZmbZIP33* resulted in an increased water loss rate and reduced drought resistance of the BMV-ZmbZIP33 due to increased stomatal opening. The results suggested that silencing the expression of *ZmbZIP33* hinders ABA accumulation and increases stomatal opening, leading to an increased water rate loss, ultimately, reducing drought resistance.

### Analyze of ABA Sensitivity of Overexpressing or Silencing *ZmbZIP33*

**Figure 7:**
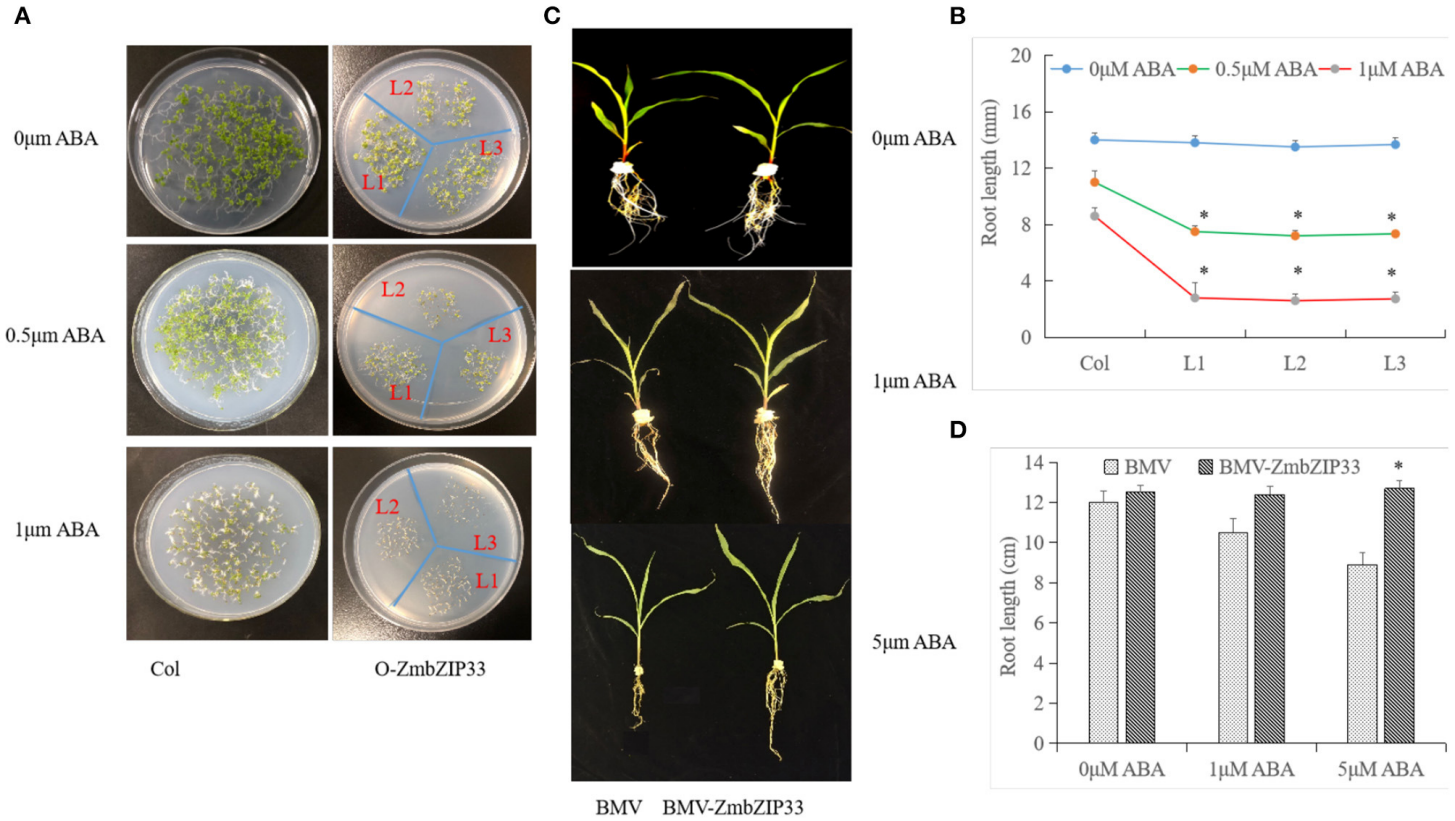
Analyze ABA sensitivity of overexpressing and silencing ZmbZIP33. **(A)** Seeds of wild-type (Col) and transgenic plants (L1–L3) germinated and were grown on MS medium with 0, 0.5, and 1 μmol/L ABA for 10 days. **(B)** Statistical analysis of the root length of transgenic *Arabidopsis* plants and Col grown on MS medium. **(C)** Three-leaf-stage of BMV and BMV-ZmbZIP33 mutant were grown in Hoagland's nutrient solution containing 0, 1 and 5 μmol/L ABA for 5 days. **(D)** Statistical analysis of the root length of BMV-ZmbZIP33 and BMV. Mean values and standard errors (bar) were shown from five independent experiments. Independent *t*-tests for equality of means demonstrated that there was significant difference between control and treatment (*P value < 0.05).

To investigate whether *ZmbZIP33* is involved in the ABA signaling pathway, transgenic and WT *Arabidopsis* lines were treated with ABA. When germinated and grown on MS medium, *ZmbZIP33* transgenic plants showed no difference in growth as compared to Col plants. However, when cultured on MS medium with 0, 0.5, and 1 μmol/L ABA for ten days, seed germination and primary root growth of *ZmbZIP33* transgenic plants (L1–L3) was severely inhibited, compared to Col (Figure 7A). The root lengths of L1, L2, and L3 transgenic lines were significantly less than those of Col at 40 – 60% for plants grown on MS medium containing 0.5 and 1 μM ABA, respectively (Figure 7B).

Sensitivity to ABA of maize lines with silenced *ZmbZIP33* was also investigated at 0, 1, and 5 μmol/L ABA for 5 days. As shown in Figure 7C, the root growth of BMV was greatly inhibited for the 1 and 5 μmol/L ABA treatments, but not for the BMV-ZmbZIP33. It indicated that silencing the expression of *ZmbZIP33* (BMV-ZmbZIP33) plants displayed ABA insensitivity. We speculated that activation of *ZmbZIP33* is important for its participation in ABA signal transduction.

### Interaction of Core Components in the ABA Signaling Pathway

**Figure 8:**
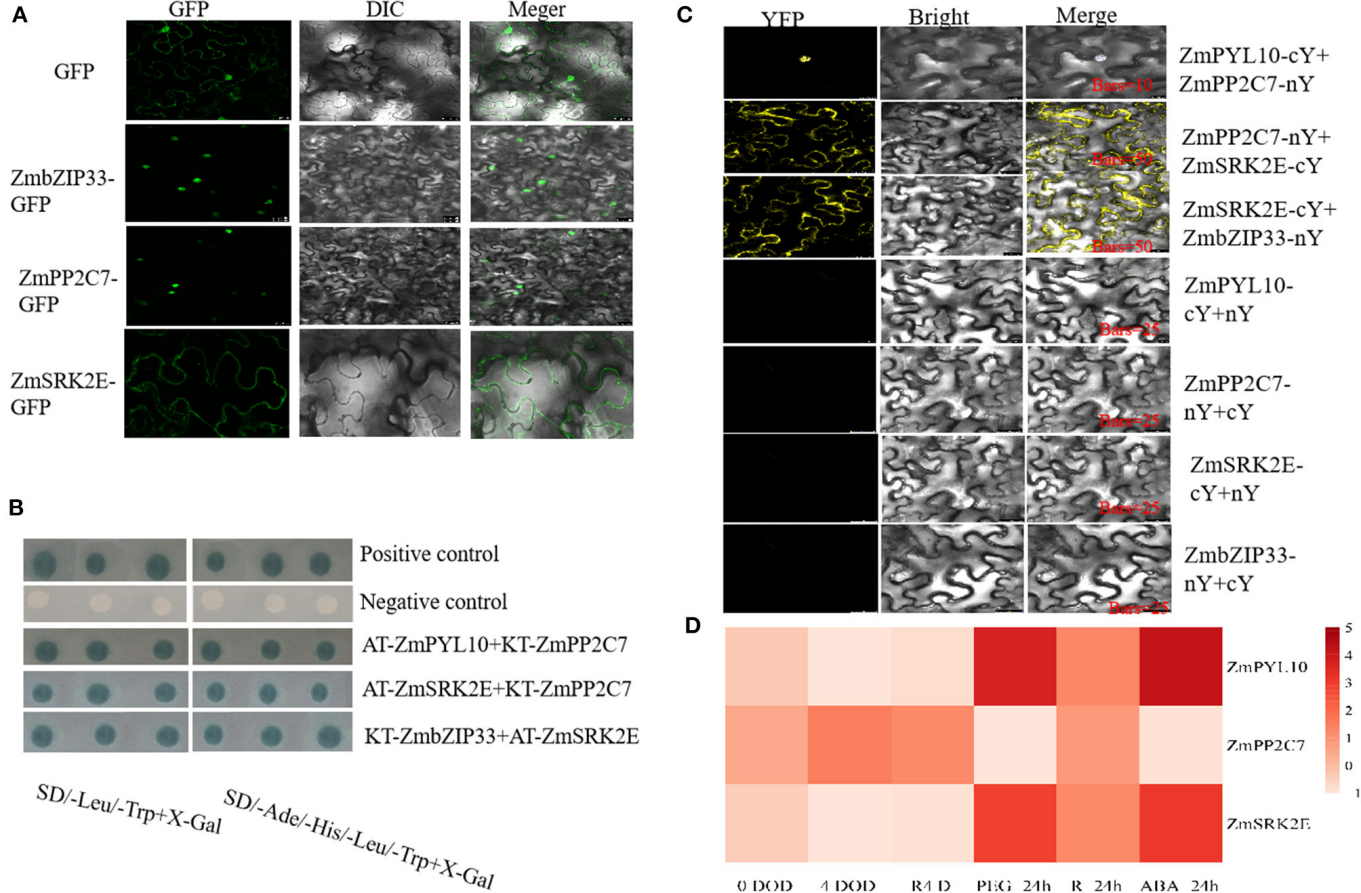
Interaction analysis of ZmbZIP33, ZmPP2C7, ZmSRK2E, and ZmPYL10 and their expression patterns under drought stress and rewateing. **(A)** Fusion proteins were transiently expressed under the control of CaMV35S promoter in tobacco leaves and were subsequently observed under a laser scanning confocal microscope. Bars = 25 μm. **(B)** The pGADT7-T+pGBKT7-Lam and pGADT7-T+pGBKT7-53, as negative and positive controls, respectively. **(C)** The nYFP and cYFP represent pSAT4-nEYFP and pSAT4-cEYFP vectors, respectively. When an interaction relationship was verified, DAPI (nuclear marker) staining was selected. The interaction signal of ZmPYL10-cY and ZmPP2C-nY coincided with DAPI, indicating an interaction within the nucleus. **(D)** The expressions of *ZmPP2C7, ZmSRK2E*, and *ZmPYL10* in BMV and BMV-ZmbZIP33 under drought stress for 0 days (0 DOD) and 4 days (4 DOD), and rewatering for 4 days (R4D) were measured. And the response patterns of *ZmPP2C7, ZmSRK2E*, and *ZmPYL10* to drought stress (PEG-24 h), rewatering (R-24 h) and ABA (ABA-24 h, 5 μmol/L) treatments for 24 h were measured. Using maize 18S as an internal control, the relative expression was determined by 2^−ΔΔCt0h^ to calculate.

The PYR/PYL receptor, PP2C protein, and SnRK2 kinase are key members of this pathway and therefore we explored the position and interaction of these core components. Pathways analysis of transcriptome differential genes found that *ZmPYL10, ZmPP2C7, ZmSRK2E*, and *ZmbZIP33* are enriched in the ABA signaling pathway. Therefore, these four genes were selected to explore the ABA signal pathways in *ZmbZIP33* transgenic lines in response to drought stress and rewatering. The subcellular localization of ZmbZIP33-GFP and ZmPP2C7-GFP were determined by observation of green fluorescence, which was found in the nucleus only, indicating that ZmbZIP33 and ZmPP2C7 are located there. By contrast, green fluorescence from ZmSRK2E-GFP was observed in the plasma membrane, indicating that ZmSRK2E is located in the plasma membrane (Figure 8A). The results of Y2H (Figure 8B) showed that co-transformation of AT-ZmPYL10 and KT-ZmPP2C7, KT-ZmPP2C7 and AT-ZmSRK2E, AT-ZmSRK2E, and KT-ZmbZIP33 all showed blue spots, indicating an interaction within each of these pairs. BIFC results showed (Figure 8C) that co-transformation of ZmPYL10-cY and ZmPP2C7-nY, ZmPP2C7-nY and ZmSRK2E-cY, ZmSRK2E-cY and ZmbZIP33-nY showed strong yellow fluorescence signals in the nucleus, plasma membrane and plasma membrane, respectively (Figure 8C). This indicated that ZmPYL10 interacts with ZmPP2C7 in the nucleus, ZmPP2C7 interacts with ZmSRK2E in the plasma membrane, and ZmSRK2E interacts with ZmbZIP33 in the plasma membrane.

To further verify whether *ZmbZIP33* responds to drought stress and rewatering through this pathway, we analyzed the expression of genes encoding these interacting proteins (Figure 8D). Expression levels were tested in BMV-ZmbZIP33 and BMV following 0 DOD, 4 DOD, and R4 D treatment as well as during 24 h treatments with PEG, rewatering, and ABA. It was found that under 0 DOD, 4 DOD, and R4 D treatments, the expression levels of *ZmPYL10* and *ZmSRK2E* in the BMV-ZmbZIP33 were lower than those of BMV, while the opposite was ture for *ZmPP2C7*. However, the expressions of *ZmPYL10* and *ZmSRK2E* in normal maize leaves were significantly up-regulated by 3–4 folds after drought stress and 24 h ABA treatment, while *ZmPP2C7* was down-regulated. These results indicated that ZmbZIP33 may form oligomers with ZmSRK2E and ZmPP2C7 in response to drought stress.

### The Expression of Genes Related to ABA Biosynthesis Is Changed in *ZmbZIP33* Transgenic Line Under Drought Stress and Rewatering

**Figure 9:**
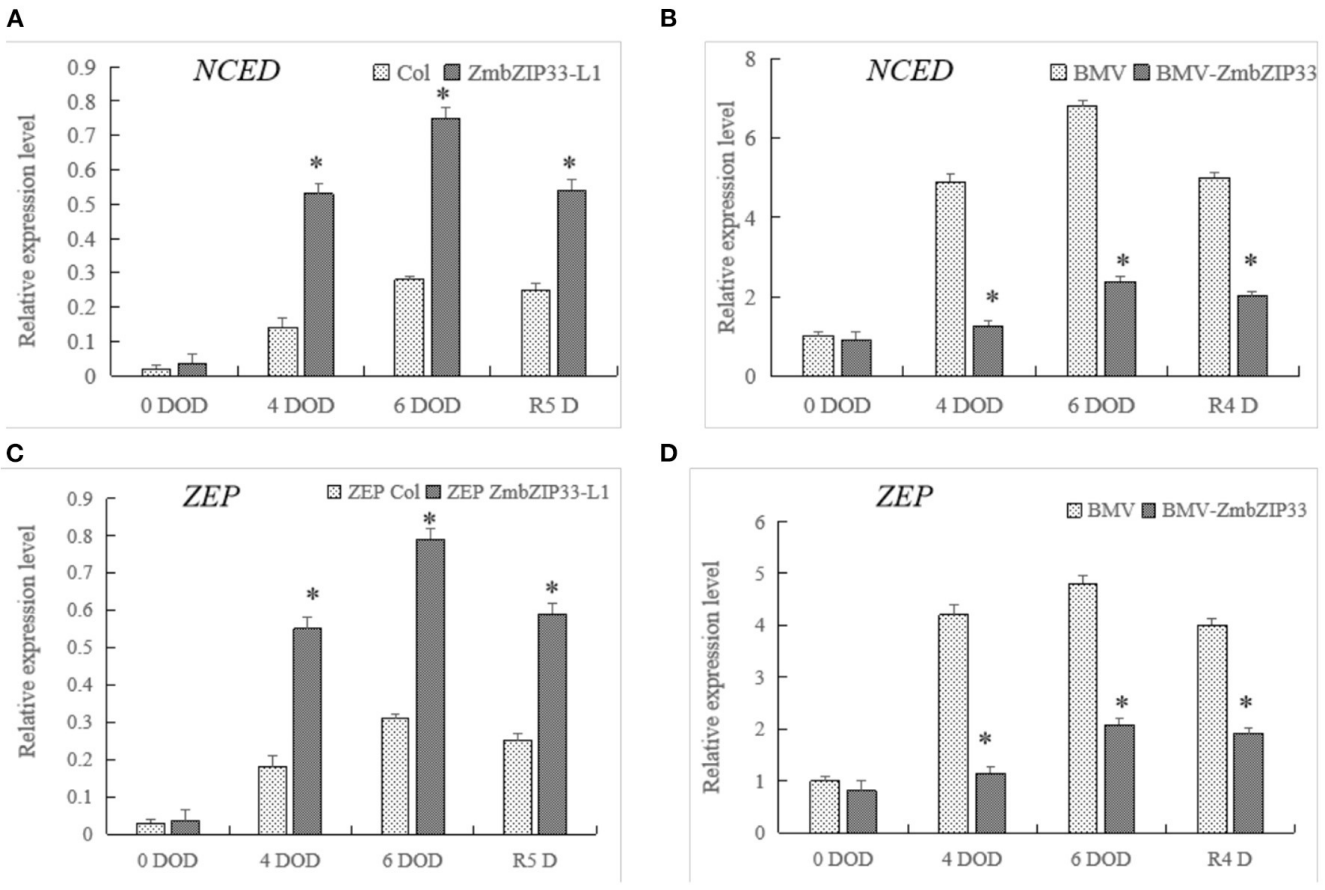
The expression of genes related to ABA synthesis in *ZmbZIP33* transgenic line and control under drought stress and rewatering. **(A,B)** *NCED-9-cis-epoxycarotenoid dioxygenase*. **(C,D)** *ZEP-Zeaxanthin epoxidase*. Mean values and standard errors (bar) were shown from five independent experiments. Independent *t*-tests for equality of means demonstrated that there was significant difference between control and treatment (*P value < 0.05).

Having shown that overexpression of *ZmbZIP33* improved the sensitivity of *Arabidopsis* to ABA during seed germination/root growth and drought-tolerant and recovery ability. We further explored whether *ZmbZIP33* responds to maize drought stress and rewatering through an ABA-dependent pathway. Therefore, we analyzed the expression of key genes for ABA biosynthesis in ZmbZIP33 transgenic line and control under drought stress and rewatering. *9-cis-epoxycarotenoid dioxygenase (NCED)* and *Zeaxanthin epoxidase (ZEP)* encode key enzymes in ABA biosynthesis. Befoer drought stress (0 DOD) there were no significant differences between the expression level of *NCED* and *ZEP* in Col and ZmbZIP33 and in BMV and BMV-ZmbZIP33 (Figure 9). Drought stress induced expression of *NCED* and *ZEP* in both genotypes, the expression level began to decrease after rewatering. Compared with the control, the expression levels of *NCED* and *ZEP* were higher in overexpression *ZmbZIP33* transgenic *Arabidopsis* under drought stress and rewatering. However, the expression of *NCED* and *ZEP* in BMV-ZmbZIP33 was significantly lower than that of BMV under drought stress and rewatering.

## Discussion

TFs are known to play an important role in many biological processes in plants, including plant development, defense regulation, and stress response. In this study, transcriptome analysis was used to screen out seventy seven TFs (MYB/MYC, bZIP, NAC, HD-ZIP, and DREB families) that respond to drought stress and rewatering in maize (Figure 1). Among them, bZIP TFs comprise a large family of regulatory proteins with various functions. In recent years, the functional analysis of bZIP gene family has been widely reported in different species, especially in *Arabidopsis* and rice (Yang et al., 2009; Yun et al., 2010; Lakra et al., 2015). In maize, more than 170 bZIP proteins have been identified (Wei et al., 2012), but the functions of only a few maize bZIP genes have been reported thus far (Ma, 2018). Gene co-expression network can identify key genes. In Cynanchum, key genes related to drought resistance have also been detected by gene co-expression network (Zhang et al., 2019). In maize, *ZmbZIP22, ZmbZIP18, ZmbZIP54*, and *ZmbZIP29*,were identified by this method. In our study, we found that *ZmbZIP33, ZmbZIP15*, and *ZmbZIP19* were the core genes in responsed to drought stress and rewatering, especially *ZmbZIP33* by gene co-expression network (Figure 2). *ZmbZIP33* encodes ABSCISIC ACID-INSENSITIVE 5-like protein 5. This family protein is mainly involved in ABA-activated signaling pathway and positive regulation of transcription (Jakoby et al., 2002). Studies have reported that the homologous proteins SbABI1 and OsABA1 of ZmbZIP33 respond to abiotic stress through the ABA-dependent pathway (Kobayashi et al., 2005). The qRT-PCR analysis showed that *ZmbZIP33* expression was up-regulated eight folds after 24 h of drought stress, and after 24 h of rewatering, the expression had returned to CK (0 h) level. After treatment with NaCl, 37°C and ABA, *ZmbZIP33* expressions were also up-regulated 2–3 folds (Figure 3). Liu and Wang confirmed that both *ZmbZIP71* and *ZmbZIP81* in maize are induced by ABA, drought and salt stress (Liu and Wang, 2011; Wang et al., 2014). These results supported our hypothesis that *ZmbZIP33* acts as an ABA-dependent regulator of abiotic stress in maize.

### Drought Resistance and Recovery Ability of *ZmbZIP33* in *Arabidopsis* and Maize

Yoshida et al. found that *AREB1, AREB2*, and *ABF3*, which encode ABSCISIC ACID-INSENSITIVE 5-like protein, are the main regulators, interacting with SnRK2.2 to regulate the expression of key genes in the ABA signaling pathway under drought stress, thereby improving the drought resistance of Arabidopsis (Yoshida et al., 2010). Kang et al. confirmed that the drought resistance of transgenic Arabidopsis plants overexpressing *ABF3, AREB2*, and *ABF4* was significantly improved, while the three mutants of *areb1, areb2*, and *abf3* were very sensitive to drought (Kang et al., 2002). Ying et al. found that overexpression of *ZmbZIP72* can significantly enhance the tolerance of *Arabidopsis* to drought (Ying et al., 2012). Zong et al. found that *OsbZIP23* plays an important role in rice drought resistance (Zong et al., 2016). Yang et al. found that overexpression of *ZmbZIP17* increased the expression of ABA-responsive genes, thereby improving the drought resistance of *Arabidopsis* (Yang et al., 2013). In our study we found that overexpression *ZmbZIP33* of *Arabidopsis* transgenic lines showed lower leaf wilting and longer roots under drought stress and rewatering compared to CK (Figures 5A–D). Growth of the root system is positively related to the ability of maizes to absorb and transport water. Therefore, overexpression of *ZmbZIP33* in transgenic *Arabidopsis* enhanced drought resistance and recovery ability. However, the drought resistance and recovery ability of BMV-ZmbZIP33 mutant are reduced, indicating that *ZmbZIP33* is a positive regulator of drought stress and rewatering (Figure 6A).

Chlorophyll is a pigment related to photosynthesis, which is responsible for the capture and transmission of light energy and the process of transforming it into chemical energy during photosynthesis. A change of chlorophyll content affects the intensity of photosynthesis. The results of this study showed that chlorophyll content of overexpressing *ZmbZIP33* transgenic *Arabidopsis* leaves was significantly higher than that of wild-type (Figure 5E). However, after 4 and 6 days drought stress and 4 days rewatering, the leaves chlorophyll content of BMV-ZmbZIP33 was significantly lower than that of control (Figure 6B). The results showed that the *ZmbZIP33* transgenic line affected the chlorophyll content of leaves under drought stress and rewatering.

Under drought stress, the accumulation of reactive oxygen species results in the peroxidation of cell membrane lipids, which leads to disruption of physiological function of maize and can even cause cell death. Antioxidant enzymes such as SOD and POD can scavenge reactive oxygen species, which is an important regulatory mechanism to resist drought stress. As the first line of defense of maize antioxidant system, SOD primarily catalyzes the dismutation of two superoxide free radicals to form oxygen and hydrogen peroxide. Following this, hydrogen peroxide reduction is catalyzed by a peroxidase which removes active oxygen. Collin et al. confirmed that increased expression of POD and SOD reduces drought-induced damages in barley (Collin et al., 2020). This study found that under drought stress and rewatering, lower SOD and POD activities were measured in the BMV-ZmbZIP33 mutant than BMV (Figures 6C,D). The results speculated that drought stress may cause more damage to the cell membrane of BMV-ZmbZIP33.

Moreover, we observed a higher ABA content in *Arabidopsis* transgenic lines than in Col under drought stress and rewatering, ABA content in *Arabidopsis* transgenic lines dropped rapidly and tended to the Col level (Figure 5F). However, ABA content in BMV-ZmbZIP33 mutant was significantly lower than that of BMV under drought stress, and after rewatering ABA content in BMV-ZmbZIP33 mutant declined rapidly to the Col level (Figure 6E). The results showed that *ZmbZIP33* may affects ABA biosynthesis or accumulation in drought and rewatering. Another physiological trait that ensures a low water loss is stomatal closure in response to water scarcity. Stomatal movement is regulated by ABA content with high ABA content promoting stomata closure (Saito and Uozumi, 2019). Under drought stress, ABA content of BMV-ZmbZIP33 mutant was lower than that of BMV, and it was found to have a higher number of open stomata numbers. After rewatering, the difference of ABA content between BMV-ZmbZIP33 and BMV was small, the stomatal state was similar (Figure 6F). Stomata affected the physiological functions of transpiration and photosynthesis. In the transpiration process, it is the main factor that directly controls the water loss. This study found that under drought stress and after rewatering, BMV-ZmbZIP33 had a significantly increased water loss rate compared to BMV (Figure 6G). This showed that *ZmbZIP33* controlled stomatal movement by regulating ABA content, which in turn affected water loss rate under drought stress and rewatering. In *Arabidopsis*, ABSCISIC ACID-INSENSITIVE 5-like protein, AtABF3 and AtABF1 take part in ABA signaling under abiotic stresses (Yoshida et al., 2015). In summary, these findings demonstrated the role of *ZmbZIP33* in ABA biosynthesis and signal transduction regulation under drought stress and rewatering.

### The Sensitivity of *ZmbZIP33* to ABA

Drought related ABA-dependent and ABA-independent pathways have been defined in plants. Most of the identified bZIP proteins were highly sensitive to ABA and can regulate abiotic stress response through ABA-dependent signaling pathway, e.g., *AtAREB, AtABFs, OsbZIP33*, and *OsbZIP23* (Xiang et al., 2008; Chen et al., 2015; Wang et al., 2017). Kang et al. confirmed that the *Arabidopsis* plants overexpressing *ABF3, AREB2*, and *ABF4* were significantly more sensitive to ABA, while the three mutants *areb1, areb2*, and *abf3* were less sensitive to ABA (Kang et al., 2002). In wheat ABSCISIC ACID-INSENSITIVE 5-like protein, TaABI5 overexpression in *Arabidopsis* caused ABA-hypersensitive (Utsugi et al., 2020). In this study, we found that overexpression of *ZmbZIP33 Arabidopsis* plants were significantly sensitivity to ABA, but silencing *ZmbZIP33* were insensitive to ABA (Figure 7). We speculated that activation of *ZmbZIP33* may be important for its participation in ABA signaling transduction.

### *ZmbZIP33* Responds to Drought Stress May Through an ABA-Dependent Signaling Pathway

From the above results, it can be seen that *ZmbZIP33* is not only significantly up-regulated after being induced by ABA induction, but also that the *ZmbZIP33* overexpressing *Arabidopsis* was more sensitive to ABA than Col, while the *ZmbZIP33*-silent maize was not sensitive to ABA. In addition, this gene regulates the ABA synthesis process to participate in drought and rewatering. Therefore, we speculate that *ZmbZIP33* responded to drought stress and rewatering through an ABA-dependent pathway. In this study, we found that ZmPYL10 interacted with ZmPP2C7 in the nucleus, ZmPP2C7 interacted with ZmSRK2E in the plasma membrane, and ZmSRK2E interacted with ZmbZIP33 in the plasma membrane (Figures 8A–C). We also analyzed the expression levels of *ZmPYL10, ZmPP2C7*, and *ZmSRK2E* in BMV and BMV-ZmbZIP33 mutant, and under drought stress, rewatering and ABA treatment. We found that under drought stress and rewatering, expression levels of *ZmPYL10* and *ZmSRK2E* in BMV-ZmbZIP33 were lower than those of BMV, while opposite was found for *ZmPP2C7*. The expression of *ZmPYL10* and *ZmSRK2E* were significantly up-regulated after drought stress and ABA treatment, while *ZmPP2C7* was down-regulated. In the ABA signaling pathway, PYR/PYL is both an ABA receptor and suppressor of PP2C. PYR/PYL interacts with PP2Cs and inhibit phosphatase activity, allowing SnRK2s positively regulates the phosphorylation of downstream ABFs genes (Sakuma et al., 2007; Ma et al., 2009; Skubacz et al., 2016). In maize, ZmbZIP4 (ABSCISIC ACID-INSENSITIVE 5-like protein family member) is a phosphorylation substrate of ZmSRK2s and participates in an ABA-dependent signal pathway (Wang et al., 2018). Zong et al. have confirmed that OsABA1 (homologous protein of ZmbZIP33) can be phosphorylated by SAPK2 and SAPK6, and participates in an ABA-dependent signal pathway (Zong et al., 2016). In Barley, overexpression of *HvABI5* increases *HvSnRK2.1* expression, enabling the amplification of ABA signaling and activation of ABA-related genes. Under drought stress, *HvABI5* directly upregulated of ABA synthesis, and ABA signaling *via* induction of *HvPP2C4* and *HvSnRK2.1* in response to drought stress (Collin et al., 2020).

*9-cis-epoxycarotenoid dioxygenase (NCED)* and *Zeaxanthin epoxidase (ZEP)* encoding key enzymes in ABA biosynthesis. In our study, *NCED*, which is a rate-limiting enzyme for ABA biosynthesis, showed a much higher expression in overexpression *ZmbZIP33* transgenic line, compared to control, in response to drought stress and rewatering. However, the expression of *NCED* in BMV-ZmbZIP33 was significantly lower than that of BMV under drought stress and rewatering. The expression of *ZEP* is the same as *NCED* (Figure 9).

Therefore, we speculate that overexpression of *ZmbZIP33* promotes ABA biosynthesis, which may induce ABA and ZmPYL10 to form a complex under drought stress. Subsequently, ZmbZIP33 may form oligomers with ZmSRK2E and ZmPP2C7, eventually inducing gene expression and stomata closure, thereby reducing water evaporation, and restoring growth in a short time. Our results provided a reliable reference for exploring how ZmPYL10, ZmPP2C7, ZmSRK2E, and ZmbZIP33 in response to drought stress through the ABA signaling pathway.

## Conclusion

This study results bring forth new data regarding the function of *ZmbZIP33* in the ABA signaling pathway under drought stress and rewatering. We found that *ZmbZIP33* positively responded to drought and rewatering by regulating chlorophyll content, antioxidant enzyme activity, ABA content, stomatal movement and leaf water loss rate. Overexpression of *ZmbZIP33* improved the sensitivity of *Arabidopsis* to ABA. The interaction of ABA core components ZmPYL10, ZmPP2C7, ZmSRK2E, and ZmbZIP33 in cells was clarified. The expression of *ZmPYL10, ZmPP2C7*, and *ZmSRK2E* genes in BMV-ZmbZIP33 mutant, and the expression patterns in response to drought, rewatering and ABA treatment were analyzed. The *NCED* and *ZEP* encoding key enzymes in ABA biosynthesis are up-regulated in overexpression *ZmbZIP33* transgenic line under drought stress and rewatering conditions, but down-regulated in BMV-ZmbZIP33 mutants.

Together, these findings revealed that the ABA-dependent regulatory role of *ZmbZIP33* under drought stress and rewatering, and increased our understanding of bZIP (ABSCISIC ACID-INSENSITIVE 5-like protein 5) response to abiotic stress though an ABA-dependent signaling pathway. It also provides a reliable reference for exploring the mechanism of ZmPYL10, ZmPP2C7, ZmSRK2E and ZmbZIP33 in response to the ABA-signaling pathway.

## Data Availability Statement

The sequencing data has been published in NCBI, and the accession number is PRJNA477643. The other original contributions presented in the study are included in the article/Supplementary Material, further inquiries can be directed to the corresponding author/s.

## Author Contributions

LC, XL, LW, TW, and ZW: conceptualization. LC, GW, QZ, XZ, and YC: methodology. GW, LC, and ZF: formal analysis. LC and XL: writing-original draft. LW, TW, and ZW: writing-review and editing. XL and ZW: funding acquisition. All authors: contributed to the article and approved the submitted version.

## Conflict of Interest

The authors declare that the research was conducted in the absence of any commercial or financial relationships that could be construed as a potential conflict of interest.
