# Supplementary material for: Maize ZmbZIP33 Is Involved in Drought Resistance and Recovery Ability Through an Abscisic Acid-Dependent Signaling Pathway
Source: Front Plant Sci. 2021 Apr 1;12:629903. doi: 10.3389/fpls.2021.629903 (PMC8048716; doi:10.3389/fpls.2021.629903)
Supplement: Supplementary file 1 [file Data_Sheet_1.doc]

Supplementary Fig.1 Phenotype of maize during drought stress and rewatering

**
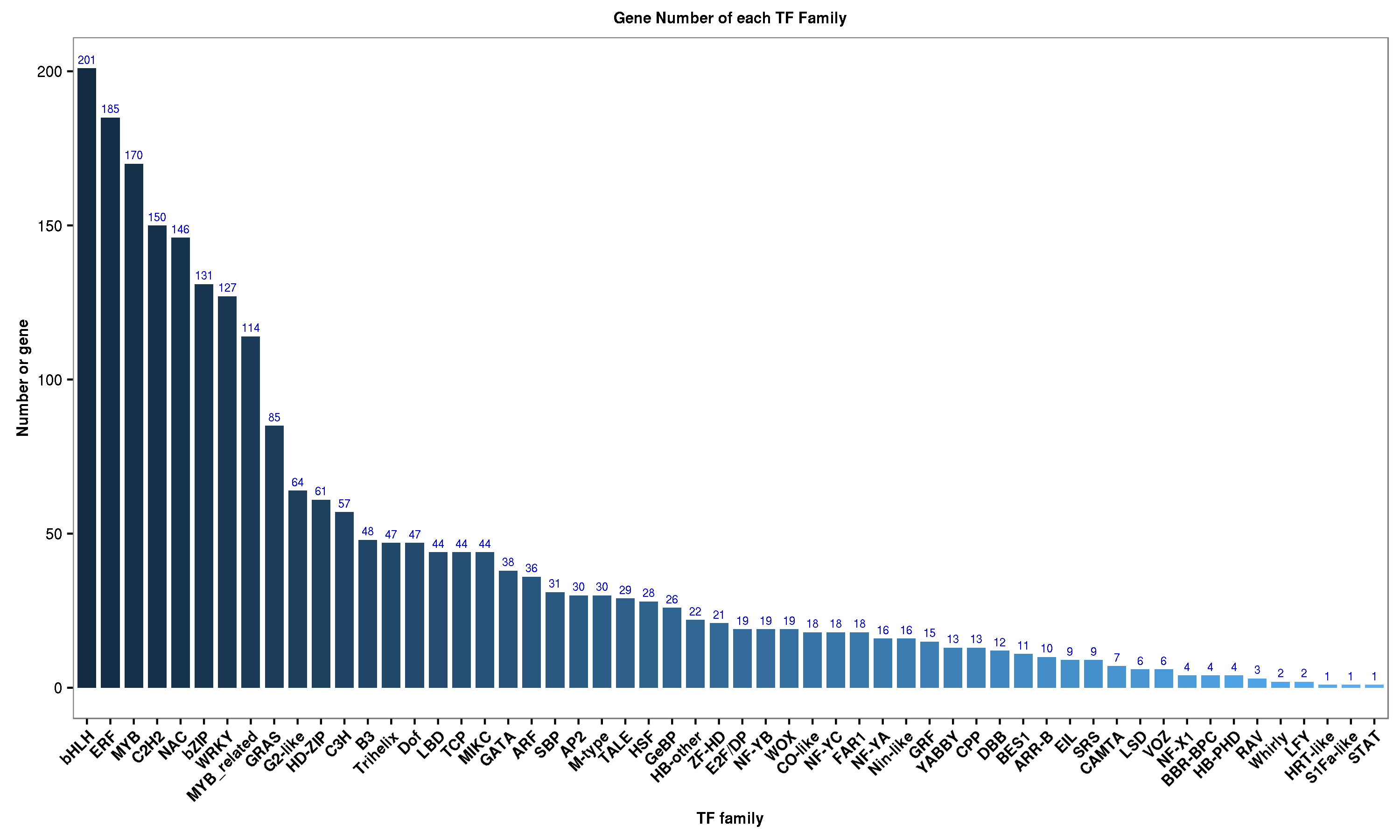
**

Supplementary Fig.2 Transcription factors identified by transcriptome sequencing

Supplemental Table S1. Expression of genes involved in ABA signaling pathway

| **ID** | **TF Family** | **log2(FC)** | **log2(FC)** | **log2(FC)** | **Pathway_ID** | **shot name** |
| --- | --- | --- | --- | --- | --- | --- |
| AC232238.2_FG004 | bZIP | -0.473 | 1.449 | 0.191 | - | ZmbZIP1 |
| GRMZM2G000171 | bZIP | -0.32 | -0.346 | 0.137 | - | ZmbZIP2 |
| GRMZM2G000842 | bZIP | 1.073 | 1.199 | 2.181 | - | ZmbZIP3 |
| GRMZM2G002075 | bZIP | 0.818 | -0.074 | 0.128 | K14432 | ZmbZIP4 |
| GRMZM2G008166 | bZIP | 0.989 | 2.507 | -1.519 | - | ZmbZIP5 |
| GRMZM2G011932 | bZIP | 0.557 | 1.574 | 0.194 | - | ZmbZIP6 |
| GRMZM2G019106 | bZIP | 0.349 | 0.399 | 0.324 | - | ZmbZIP7 |
| GRMZM2G025812 | bZIP | -0.879 | 0.221 | -0.752 | - | ZmbZIP8 |
| GRMZM2G030280 | bZIP | -0.014 | 0.272 | 0.159 | K14431 | ZmbZIP9 |
| GRMZM2G033230 | bZIP | 1.4 | 1.93 | 0.151 | - | ZmbZIP10 |
| GRMZM2G033413 | bZIP | 0.612 | 0.887 | 0.642 | - | ZmbZIP11 |
| GRMZM2G045236 | bZIP | 0.363 | 0.17 | -0.512 | - | ZmbZIP12 |
| GRMZM2G050912 | bZIP | 0.079 | 1.337 | -0.464 | - | ZmbZIP13 |
| GRMZM2G055413 | bZIP | -0.266 | -0.008 | -0.115 | - | ZmbZIP14 |
| GRMZM2G056099 | bZIP | 0.141 | 0.259 | -0.112 | K14431 | ZmbZIP15 |
| GRMZM2G060290 | bZIP | 0.343 | 0.62 | 0.134 | K14431 | ZmbZIP16 |
| GRMZM2G066734 | bZIP | 2.485 | 0.075 | -1.582 | - | ZmbZIP17 |
| GRMZM2G073892 | bZIP | -0.287 | -0.468 | 0.597 | - | ZmbZIP18 |
| GRMZM2G079365 | bZIP | 0.054 | 0.287 | -0.672 | - | ZmbZIP19 |
| GRMZM2G088140 | bZIP | 0.113 | 0.5 | -0.127 | - | ZmbZIP20 |
| GRMZM2G112483 | bZIP | -0.447 | -0.294 | -1 | - | ZmbZIP21 |
| GRMZM2G118870 | bZIP | -0.259 | 0.039 | -0.419 | - | ZmbZIP22 |
| GRMZM2G120167 | bZIP | -0.159 | 0.126 | 0.375 | - | ZmbZIP23 |
| GRMZM2G122846 | bZIP | 0.543 | 0.66 | -1.314 | - | ZmbZIP24 |
| GRMZM2G125243 | bZIP | 0.296 | 0.446 | -0.904 | - | ZmbZIP25 |
| GRMZM2G129247 | bZIP | 0.46 | 0.701 | -0.144 | K14432 | ZmbZIP26 |
| GRMZM2G132868 | bZIP | 0.669 | 0.339 | -0.208 | K14432 | ZmbZIP27 |
| GRMZM2G133331 | bZIP | 0.492 | -0.641 | 0.212 | - | ZmbZIP28 |
| GRMZM2G134863 | bZIP | -0.169 | -0.465 | -0.793 | - | ZmbZIP29 |
| GRMZM2G136266 | bZIP | 0.406 | 0.162 | -0.342 | - | ZmbZIP30 |
| GRMZM2G149150 | bZIP | 0.749 | -0.324 | -1.918 | - | ZmbZIP31 |
| GRMZM2G157722 | bZIP | 1.124 | 1.624 | -0.783 | K14432 | ZmbZIP33 |
| GRMZM2G171912 | bZIP | -1.011 | 1.98 | -0.123 | - | ZmbZIP32 |
| GRMZM2G336766 | bZIP | 0.389 | 1.659 | -2.472 | - | ZmbZIP34 |
| GRMZM2G361611 | bZIP | -1.406 | -1.119 | 1.495 | - | ZmbZIP35 |
| GRMZM2G425920 | bZIP | 0.346 | 0.486 | -0.208 | K16241 | ZmbZIP36 |
| GRMZM2G438293 | bZIP | 0.498 | 0.467 | -0.649 | - | ZmbZIP37 |
| GRMZM2G444748 | bZIP | 0.004 | -0.093 | 0.165 | - | ZmbZIP38 |
| GRMZM2G445575 | bZIP | 0.124 | 0.78 | 0.271 | - | ZmbZIP39 |
| GRMZM2G448607 | bZIP | -0.653 | -0.583 | 0.273 | - | ZmbZIP40 |
| GRMZM5G858197 | bZIP | 0.47 | 1.151 | -0.871 | - | ZmbZIP41 |
| XLOC_045567 | MYB | 0.056 | -0.477 | 0.141 | K14432 | ZmMYB1 |
| GRMZM5G889719 | DREB | -1.466 | -0.866 | -3.358 | - | ZmDREB1 |
| AC187157.4_FG005 | HD-Zip | 2.641 | 1.494 | -0.995 | - | ZmZIP1 |
| GRMZM2G469551 | HD-Zip | 1.273 | 1.447 | -1.107 | - | ZmZIP3 |
| XLOC_025615 | HD-Zip | -0.451 | -0.154 | 1.008 | - | ZmZIP2 |
| AC206901.3_FG005 | MYB | 1.128 | -1.112 | -4.191 | - | ZmMYB2 |
| GRMZM2G002128 | MYB | -0.711 | -0.377 | 0.509 | - | ZmMYB3 |
| GRMZM2G022686 | MYB | -0.654 | 0.517 | 0.084 | - | ZmMYB4 |
| GRMZM2G047626 | MYB | -1.771 | -0.664 | -0.954 | - | ZmMYB5 |
| GRMZM2G050305 | MYB | 0.287 | 1.278 | -0.335 | - | ZmMYB6 |
| GRMZM2G051256 | MYB | -0.502 | -0.04 | -3.108 | - | ZmMYB7 |
| GRMZM2G078820 | MYB | 1.628 | -0.311 | -3.539 | - | ZmMYB8 |
| GRMZM2G096358 | MYB | 1.849 | 0.633 | -1.84 | - | ZmMYB9 |
| GRMZM2G098179 | MYB | -1.506 | 1.109 | -2.728 | - | ZmMYB10 |
| GRMZM2G145444 | MYB | -1.567 | -2.565 | -2.73 | - | ZmMYB11 |
| GRMZM2G169316 | MYB | 0.231 | -0.219 | -0.277 | - | ZmMYB12 |
| GRMZM5G803308 | MYB | 0.047 | -0.376 | -1.918 | - | ZmMYB13 |
| AC196475.3_FG005 | NAC | 0.205 | 0.02 | -1.16 | - | ZmNAC1 |
| AC203535.4_FG002 | NAC | 0.486 | 0.645 | -1.021 | - | ZmNAC2 |
| AC212859.3_FG008 | NAC | -1.796 | -0.344 | 1.227 | - | ZmNAC3 |
| GRMZM2G003715 | NAC | 0.64 | -0.132 | -1.84 | - | ZmNAC4 |
| GRMZM2G042494 | NAC | 2.956 | -0.678 | -8.835 | - | ZmNAC5 |
| GRMZM2G068973 | NAC | -0.961 | -1.902 | -1.937 | - | ZmNAC6 |
| GRMZM2G074358 | NAC | -0.978 | -1.8 | -2.078 | - | ZmNAC7 |
| GRMZM2G081930 | NAC | 3.806 | 4.69 | -5.119 | - | ZmNAC8 |
| GRMZM2G109627 | NAC | 1.589 | 0.374 | -1.732 | - | ZmNAC9 |
| GRMZM2G123667 | NAC | 0.023 | -0.005 | -1.773 | - | ZmNAC10 |
| GRMZM2G126936 | NAC | -0.353 | 0.084 | -8.261 | - | ZmNAC11 |
| GRMZM2G167018 | NAC | 0.668 | -1.626 | 1.42 | - | ZmNAC12 |
| GRMZM2G167492 | NAC | -0.151 | 0.448 | -1.128 | - | ZmNAC13 |
| GRMZM2G171395 | NAC | -1.968 | 3.511 | -3.005 | - | ZmNAC14 |
| GRMZM2G180328 | NAC | 1.094 | 2.322 | -3.015 | - | ZmNAC15 |
| GRMZM2G181605 | NAC | 0.931 | -2.767 | -2.654 | - | ZmNAC16 |
| GRMZM2G336533 | NAC | 0.425 | -0.659 | -3.915 | - | ZmNAC17 |
| GRMZM2G347043 | NAC | 0.016 | 0.303 | -1.039 | - | ZmNAC18 |
| GRMZM2G430849 | NAC | 2.84 | 1.132 | -1.793 | - | ZmNAC19 |
| GRMZM2G439903 | NAC | -0.628 | -2.945 | -2.385 | - | ZmNAC20 |

Supplemental Table S2. Primer sequences for constructing cloning vectors

| **Primer names** | **Primer sequences(5’-3’)** | **Function** |
| --- | --- | --- |
| ZmbZIP33-F | CATTACTGACCGCCTGAATCG | qRT-PCR |
| ZmbZIP33-R | AGATGACGCTGGAGGAGTTC | qRT-PCR |
| ZmbZIP33-F-AscI | AGGCGCGCCATGGATCTCAACGAAT | Transform |
| ZmbZIP33-R-BamHI | CGGGATCCTTACCAGGGACCTGTCA | Transform |
| ZmbZIP33-GFP-SpeI-F | GGACTAGT ATGGATCTCAACGAAT | SL |
| ZmbZIP33-GFP-BamHI-R | CGGGATCC TTACCAGGGACCTGTCA | SL |
| ZmSRK2E-GFP-SpeI-F | GGACTAGT ATGGAGGAGAGGTACGAG | SL |
| ZmSRK2E-GFP-BamHI-R | CGGGATCC TCAGTAGGTGTCATCGGCGTC | SL |
| ZmPP2C7-GFP-SpeI-F | GGACTAGT ATGGAGGACCTCGCCCCG | SL |
| ZmPP2C7-GFP- BamHI-R | CGGGATCC TTATGTTCTGCTCTTGAACTTTCT | SL |
| ZmbZIP33-KT-ECORI-F | TCATATGGCCATGGAGGCCAGTGAATTCATGGATCTCAACGAATGC | YTH |
| ZmbZIP33-KT- BamHI-R | ATCTGCAGCTCGAGCTCGATGGATCCTTACCAGGGACCTGTCAATG | YTH |
| ZmSRK2E-AT- ECORI-F | TCATATGGCCATGGAGGCCAGTGAATTCATGGAGGAGAGGTACGAG | YTH |
| ZmSRK2E-AT- BamHI-R | ATCTGCAGCTCGAGCTCGATGGATCCTTATGTTCTGCTCTTGAAC | YTH |
| ZmPP2C7-KT-ECORI-F | TCATATGGCCATGGAGGCCAGTGAATTCATGGAGGACCTCGCCCCG | YTH |
| ZmPP2C7-KT- BamHI-R | ATCTGCAGCTCGAGCTCGATGGATCCTCAGTAGGTGTCATCGGCGT | YTH |
| ZmPYL10-AT- ECORI-F | TCATATGGCCATGGAGGCCAGTGAATTCATGGAAGACGTCGTAGCAGT | YTH |
| ZmPYL10-AT- BamHI-R | ATCTGCAGCTCGAGCTCGATGGATCCTCAGTTTTGCTCTTGAACTTG | YTH |
| ZmbZIP33-YN- ECORI-F | AGATCTCGAGCTCAAGCTTCGAATTCATGGATCTCAACGAATGC | BIFC |
| ZmbZIP33-YN- KPNI-R | CAGGATCCCGGGCCCGCGGTACCGCCAGGGACCTGTCAATGTT | BIFC |
| ZmSRK2E-YC- ECORI-F | AGATCTCGAGCTCAAGCTTCGAATTCATGGAGGAGAGGTACGAG | BIFC |
| ZmSRK2E-YC-KPNI-R | CAGGATCCCGGGCCCGCGGTACCGGTAGGTGTCATCGGCGTCTGC | BIFC |
| ZmPP2C7-YN- ECORI-F | AGATCTCGAGCTCAAGCTTCGAATTCATGGAGGACCTCGCCCCG | BIFC |
| ZmPP2C7-YN- KPNI-R | CAGGATCCCGGGCCCGCGGTACCGTGTTCTGCTCTTGAACTTTC | BIFC |
| ZmPYL10-YC- ECORI-F | AGATCTCGAGCTCAAGCTTCGAATTCATGGAAGACGTCGTAGCAGT | BIFC |
| ZmPYL10-YC- KPNI-R | CAGGATCCCGGGCCCGCGGTACCGAGTTTTGCTCTTGAACTTG | BIFC |
| ZmbZIP33-NCOI-F | CATGCCATGGGGAGATCCGAGAGGAAGT | VIGS |
| ZmbZIP33- AvrII-R | CTAG CCTAGGGTTGTCACTGCTCTCCCT | VIGS |

| ZmSRK2E-Q-F | GAGGAGAGGTACGAG | qRT-PCR |
| --- | --- | --- |
| ZmSRK2E-Q-R | TATGTTCTGCTCTTGAAC | qRT-PCR |
| ZmPP2C7-Q-F | GGAGGACCTCGCCCCG | qRT-PCR |
| ZmPP2C7-Q-R | TCCTCAGTAGGTGTCATCGGCGT | qRT-PCR |
| ZmPYL10-Q-F | GAAGACGTCGTAGCAGT | qRT-PCR |
| ZmPYL10-Q-R | TCAGTTTTGCTCTTGAACTTG | qRT-PCR |
| NCED-F | CGACAAGTTCATCTACGGCG | qRT-PCR |
| NCED-R | CGTTGACGACGAGCATTTCC | qRT-PCR |
| ZEP-F | TTGGTTGGTGCCGATGGAAT | qRT-PCR |
| ZEP-R | CGTATCGATATCGGGCAGCA | qRT-PCR |

Note: SL stands for subcellular localization, YTH stands for yeast two-hybrid, and BIFC stands for bimolecular fluorescence complementation.


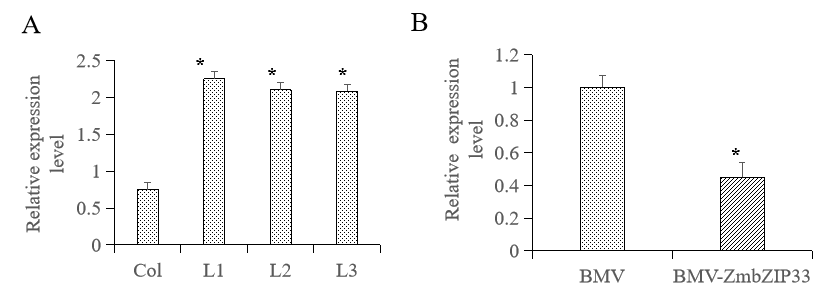


Supplementary Fig.3.The expression level of *ZmbZIP33* in transgenic lines. (A) Analysis of *ZmbZIP33* expression in transgenic lines and Col is wild type Arabidopsis, L1 ~ L3 are transgenic Arabidopsis lines.(B) Analysis of *ZmbZIP33* expression in BMV (WT) and BMV-ZmbZIP33 transgenic line.

**Supplementary Table S3. T1 segregation of bar and** bZIP33 transgenes and test goodness of fit for Medellian patterns

| **Events** | **Genes** | **Positive** | **Negative** | **Total seeds** | **Chi-square** |
| --- | --- | --- | --- | --- | --- |
| *bZIP1-1* (L1) | *bZIP33* | 100 | 35 | 135 | 0.06173 |
|  | *bar* | 95 | 40 | 135 | 1.54321 |
| *bZIP1-3* | *bZIP33* | 98 | 73 | 171 | 28.53996 |
|  | *bar* | 113 | 58 | 171 | 7.25341 |
| *bZIP2-2* | *bZIP33* | 66 | 40 | 106 | 9.16981 |
|  | *bar* | 70 | 36 | 106 | 4.54088 |
| *bZIP2-5* (L2) | *bZIP33* | 200 | 60 | 260 | 0.51282 |
|  | *bar* | 190 | 70 | 260 | 0.51282 |
| *bZIP2-7* | *bZIP33* | 53 | 56 | 109 | 40.44343 |
|  | *bar* | 70 | 39 | 109 | 6.75535 |
| *bZIP2-10* | *bZIP33* | 63 | 60 | 123 | 37.09756 |
|  | *bar* | 81 | 42 | 123 | 5.48780 |
| *bZIP3-1* | *bZIP33* | 64 | 40 | 104 | 10.05128 |
|  | *bar* | 68 | 36 | 104 | 5.12821 |
| *bZIP3-3* (L3) | *bZIP33* | 91 | 38 | 129 | 1.36693 |
|  | *bar* | 98 | 31 | 129 | 0.06460 |
